# CIN-like TCP13 is essential for plant growth regulation under dehydration stress

**DOI:** 10.1007/s11103-021-01238-5

**Published:** 2022-01-20

**Authors:** Kaoru Urano, Kyonoshin Maruyama, Tomotsugu Koyama, Nathalie Gonzalez, Dirk Inzé, Kazuko Yamaguchi-Shinozaki, Kazuo Shinozaki

**Affiliations:** 1 Gene Discovery Research Group, RIKEN Center for Sustainable Resource Science (CSRS), 3-1-1 Koyadai, Tsukuba, Ibaraki 305-0074 Japan; 2 Present Address: Institute of Agrobiological Sciences, NARO 3-1-3 Kannondai, Tsukuba, Ibaraki 305-8604 Japan; 3 Plant Biotechnology Division, Japan International Research Center for Agricultural Sciences (JIRCAS), 1-1 Ohwashi, Tsukuba, Ibaraki 305-8686 Japan; 4 Bioorganic Research Institute, Suntory Foundation for Life Sciences, Seikacho, Kyoto 619-0284 Japan; 5 INRAE, Université de Bordeaux, UMR1332 Biologie du Fruit Et Pathologie, 33882 Villenave d’Ornon Cedex, France; 6 Department of Plant Biotechnology and Bioinformatics, Ghent University, 9052 Ghent, Belgium; 7 VIB Center for Plant Systems Biology, 9052 Ghent, Belgium; 8 Laboratory of Plant Molecular Physiology, Graduate School of Agricultural and Life Sciences, The University of Tokyo, Bunkyo-ku, Tokyo, 113-8657 Japan

**Keywords:** Dehydration stress response, TCP transcription factor, Leaf morphology, Root growth, Drought tolerance

## Abstract

**Key message:**

A dehydration-inducible Arabidopsis *CIN-like TCP* gene, *TCP13*, acts as a key regulator of plant growth in leaves and roots under dehydration stress conditions.

**Abstract:**

Plants modulate their shape and growth in response to environmental stress. However, regulatory mechanisms underlying the changes in shape and growth under environmental stress remain elusive. The CINCINNATA (CIN)-like TEOSINTE BRANCHED1/CYCLOIDEA/PCF (TCP) family of transcription factors (TFs) are key regulators for limiting the growth of leaves through negative effect of auxin response. Here, we report that stress-inducible CIN-like TCP13 plays a key role in inducing morphological changes in leaves and growth regulation in leaves and roots that confer dehydration stress tolerance in *Arabidopsis thaliana*. Transgenic Arabidopsis plants overexpressing *TCP13* (*35Spro::TCP13OX*) exhibited leaf rolling, and reduced leaf growth under osmotic stress. The *35Spro::TCP13OX* transgenic leaves showed decreased water loss from leaves, and enhanced dehydration tolerance compared with their control counterparts. Plants overexpressing a chimeric repressor domain SRDX-fused TCP13 (*TCP13pro::TCP13SRDX*) showed severely serrated leaves and enhanced root growth. Transcriptome analysis of *TCP13pro::TCP13SRDX* transgenic plants revealed that TCP13 affects the expression of dehydration- and abscisic acid (ABA)-regulated genes. TCP13 is also required for the expression of dehydration-inducible auxin-regulated genes, *INDOLE-3-ACETIC ACID5* (*IAA5*) and *LATERAL ORGAN BOUNDARIES* (*LOB) DOMAIN 1* (*LBD1*). Furthermore, *tcp13* knockout mutant plants showed ABA-insensitive root growth and reduced dehydration-inducible gene expression. Our findings provide new insight into the molecular mechanism of CIN-like TCP that is involved in both auxin and ABA response under dehydration stress.

**Supplementary Information:**

The online version contains supplementary material available at 10.1007/s11103-021-01238-5.

## Introduction

Plants are exposed to various environmental stresses, such as drought, high salt, and low temperature. To withstand these abiotic stresses, plants have evolved numerous mechanisms of adaptation. Inhibition of shoot growth under water deficit conditions is often observed, which improves water balance and stress tolerance, thus ensuring plant survival (Claeys and Inze 2013). Environmental cues such as water and nutrient availability, salt, temperature, and light conditions have profound effects on leaf and root architecture (Ding and De Smet 2013). Morphological changes in leaves and roots allow plants to acclimate to and tolerate environmental stress conditions. When the effect of environmental stress is temporary, continued restriction of growth can lead to a competitive disadvantage and unnecessary yield losses. Therefore, plants have evolved several tightly regulated mechanisms to balance growth and survival in response to severe abiotic stress conditions. Although the regulation of shoot and root growth under water deficit conditions is important for plant survival, the molecular mechanisms regulating morphological changes in these organs are not well understood.

Plants utilize transcriptional modulation to regulate the balance between growth and survival under abiotic stress conditions. Previous studies have shown that genes performing different functions are either upregulated or downregulated under stress conditions (Dubois et al. 2013; Kreps et al. 2002; Maruyama et al. 2004, 2009; Seki et al. 2002a, 2002b; Urano et al. 2017, 2009). During evolution, the number of transcription factors (TFs) involved in the adaptation of plants to complex environmental stresses has increased. Several different TF families are known to be involved in abiotic stress responses, including dehydration-responsive element-binding (DREB) protein, basic leucine zipper (bZIP) domain, ethylene-responsive element-binding factor (ERF), zinc-finger, WRKY, MYB, and basic helix-loop-helix (bHLH) families. These TFs mainly function as transcriptional activators of downstream genes involved in stress responses and tolerance (Chen et al. 2002; Kodaira et al. 2011; Schommer et al. 2008; Yamaguchi-Shinozaki and Shinozaki 2006). Recently, the results of high-throughput techniques, such as chromatin immunoprecipitation sequencing (ChIP-seq) and DNA affinity purification sequencing (DAP-seq), showed that differential binding of multiple TFs to downstream gene promoters ensures robust responsiveness of downstream genes to the environmental stimulus (O'Malley et al. 2016; Song et al. 2016; Sullivan et al. 2014).

The CINCINNATA-like (CIN-like) TEOSINTE BRANCHED1/CYCLOIDEA/PCF (TCP) TF family plays essential roles in the determination of leaf size and shape (Nath et al. 2003; Palatnik et al. 2003). TCPs harbor a conserved noncanonical bHLH domain, which mediates their binding to DNA or interaction with other proteins (Cubas et al. 1999; Kosugi and Ohashi 2002). The TCP proteins are grouped into two subclasses, class I and class II, based on sequence similarity (Martin-Trillo and Cubas 2010). A total of 13 class I and 11 class II TCPs have been identified in *Arabidopsis thaliana*. Among the 11 class II *TCP* genes, *TCP2*, *TCP3*, *TCP4*, *TCP5*, *TCP10*, *TCP13*, *TCP17*, and *TCP24* belong to the *CIN-like TCP* family. Simultaneous disruption of multiple *CIN-like TCP* genes greatly affects leaf development (Koyama et al. 2010; Schommer et al. 2008). TCP3 directly activates the expression of *microRNA164* (*miR164*), *ASYMMETRIC LEAVES1* (*AS1*), *INDOLE-3-ACETIC ACID3/SHORT HYPOCOTYL2* (*IAA3/SHY2*), and several auxin-inducible genes including SMALL AUXIN UP RNA (*SAUR*) proteins, *PIN FORMED* (*PIN*) family of auxin efflux carriers, and LATERAL ORGAN BOUNDARIES (*LOB) DOMAIN* (*LBD*) TFs (Koyama et al. 2010). These target genes of TCP3 act as negative regulators of *CUP-SHAPED COTYLEDON* (*CUC*) genes for regulating leaf differentiation (Koyama et al. 2010). TCP4 directly activates the expression of *HAT2*, a HD-ZIP II TF to induce the maturation of leaf pavement cells via both auxin-dependent and independent pathways (Challa et al. 2019). TCP5 controls leaf margin development by regulating *KNAT3*, a *KNOTTED1*-like homeobox (*KNOX*) gene and *SAW1*, a BEL-like transcription factors (Yu et al. 2020). *CIN-like TCP* genes also regulate jasmonic acid (JA) and flavonol biosynthesis. JA biosynthesis is mediated by *Lipoxygenase2* (*LOX2*), which is upregulated by *TCP3* (Koyama et al. 2007) and *TCP4* (Schommer et al. 2008), and downregulated in *jaw-d* mutants (Schommer et al. 2008). TCP3 also interacts with the R2R3-MYB protein MYB12, which promotes flavonoid biosynthesis and represses auxin signaling (Li and Zachgo 2013). In addition, *CIN-like TCP* genes are involved in the control of axillary bud outgrowth. The *jaw‐D, tcp5* and *tcp5/13/17* mutant plants show a significant reduction in the number of secondary branches (van Es et al. 2019).

The activity of five *CIN-like TCPs*, including *TCP2*, *TCP3*, *TCP4*, *TCP10*, and *TCP24*, during leaf development is tightly regulated by miR319 at the post-transcriptional level. Expression of *TCPs* carrying synonymous mutations, responsible for the resistance to miR319-mediated cleavage, causes severe defects in leaf morphology or seedling death in Arabidopsis, and the transition from compound leaves to simple leaves in tomato (*Solanum lycopersicum*) (Ori et al. 2007; Palatnik et al. 2003, 2007). The activity of CIN-like TCPs is also modulated at the protein level through protein–protein interactions. ARMADILLO BTB ARABIDOPSIS PROTEIN1 (ABAP1) interacts with TCP24 to modify its activity for the regulation of leaf cell proliferation (Masuda et al. 2008). The SWI/SNF chromatin remodeling ATPase, BRAHMA (BRM), modulates the activity of CIN-like TCPs to reduce the cytokinin sensitivity of leaves by increasing the expression of the negative cytokinin regulator, *ARABIDOPSIS RESPONSE REGULATOR16* (*ARR16*) (Efroni et al. 2013). The TCP INTERACTOR-CONTAINING EAR MOTIF PROTEIN1 (TIE1), a transcriptional repressor, as well as TOPLESS (TPL) and TIE1-ASSOCIATED RING-TYPE E3 LIGASE1 (TEAR1) regulate leaf development by physically interacting with CIN-like TCPs (Tao et al. 2013; Zhang et al. 2017). However, the role of CIN-like TCP in the regulation of plant growth under abiotic stress conditions is not well characterized.

In this study, we show that an *Arabidopsis CIN-like TCP* gene, *TCP13*, is significantly induced by dehydration. We show that TCP13 plays important roles in regulating the growth of leaves and roots under abiotic stress. Arabidopsis plants overexpressing *TCP13* (*35Spro::TCP13OX*) exhibited leaf rolling and leaf growth inhibition, abscisic acid (ABA)-sensitive root growth, and elevated dehydration stress tolerance. Additionally, *tcp13* knockout mutant plants showed ABA-insensitive root growth and reduced dehydration-inducible gene expression, which supports the idea that *TCP13* acts downstream of ABA signaling pathway under dehydration stress. TCP13 also positively regulates the dehydration-inducible auxin-regulated genes, *IAA*5 and *LBD1*. Overall, our results suggest that TCP13 is an important component of a regulatory module that controls plant growth under dehydration stress.

## Materials and methods

### Plant materials and stress treatments

Arabidopsis thaliana ecotype Columbia (Col-0; WT) was used for the generation of transgenic lines used in this study. Unless otherwise stated, plants were grown on MS medium (Murashige and Skoog 1962), supplemented with 3% sucrose and 0.8% agar (MS-agar), or in soil (Dio Professional for grafting, Innovex Co., Ltd., Tokyo) at 22 °C under long-day conditions (a 16 h light/8 h dark photoperiod and 60 ± 10 μmol photons m^−2^ s^−1^ light intensity, as described previously (Urano et al. 2009; Urano et al. 2004)). The T-DNA insertion *tcp5* (SM_3_29639), *tcp13* (GK_182B12), and *tcp17* (SALK_148580) mutant lines were obtained from the Arabidopsis Biological Resource Center (ABRC), OH, USA, and the site of T-DNA insertion site in each of these mutants has been described previously (Koyama et al. 2007, 2010). The *tcp13tcp15tcp17* triple mutant was generated by crossing the single mutants described previously (Koyama et al. 2017). To perform ABA or salt (NaCl) stress treatments, 2-week-old whole WT plants were transferred from the agar medium to water (control) or to water containing 10 μM ABA or175 mM NaCl for 3–6 h. To induce dehydration stress, whole plants were transferred on to a parafilm (Parafilm M PM999, Bemis Company, Neenah, WI) for 3–6 h (WT) or 3–8 h (*tcp*13 and WT).

### Plasmid construction and plant transformation

To generate *35Spro::TCP13OX*, *35Spro::TCP5OX*, and *35Spro::TCP17OX* constructs, coding sequences (CDSs) of *TCP13*, *TCP5*, and *TCP17*, respectively, were amplified by PCR using sequence-specific primers and cloned into the *Eco*RV site of *pGKX*, as described previously (Maruyama et al. 2009). To generate the *35Spro::TCP13SRDX* and *35Spro::TCP13-sGFP* constructs, the CDS of *TCP13* minus the stop codon was cloned into the *Sma*I site of *pGKX-SRDX* and *pGKX-sGFP*, respectively, as described previously (Fujita et al. 2005; Maruyama et al. 2009; Qin et al. 2008). To construct the *TCP13pro::TCP13SRDX* plasmid, the *TCP13* promoter (∼ 1000 bp upstream of the transcription start site) was PCR amplified and cloned into *35Spro::TCP13SRDX* between *Kpn*I and *Xba*I restriction sites*.* To construct the *TCP13pro::GUS* plasmid, first the *pGKX-GUS* construct was generated by cloning the *GUS* reporter gene amplified from the *pBI101* vector (Fujita et al. 2005; Maruyama et al. 2009; Qin et al. 2008; Urano et al. 2004) into the *pGKX-sGFP* plasmid between *Bam*HI and *Ec*oRV restriction sites. Then, the *TCP13* promoter region was cloned into the *pGKX-GUS* plasmid between *Kpn*I and *Xba*I restriction sites. These constructs were introduced into *Agrobacterium tumefaciens* strain C58, which was then used to transform Arabidopsis plants via vacuum infiltration (Bechtold and Pelletier 1998). Seeds of T2 or T3 plants were used for subsequent experiments.

### Histochemical GUS staining and GFP detection in protoplasts

Histochemical GUS staining was performed as described previously (Urano et al. 2004), and GUS was observed using an M205C stereomicroscope equipped with a DFC490 digital color camera (Leica Microsystems). To determine the subcellular localization of TCP13, plasmid DNA (5 μg) isolated from *35Spro::TCP13-sGFP* plants was transfected into Arabidopsis mesophyll protoplasts, and GFP was detected under a confocal laser scanning microscope (LSM510; Zeiss). Arabidopsis protoplast isolation and PEG-calcium transfection were performed as described previously (Yoo et al. 2007).

### ABA sensitivity of transgenic and mutant plants

The ABA sensitivity test was performed as described previously (Yoshida et al. 2010) with minor modification. *35Spro::TCP13OX* and control plants were grown in plates containing 1/2 MS medium supplemented with 1% sucrose, 30 mg/L kanamycin, and 0.8% agar or *tcp13* mutant and WT (Col-0) plants were grown in plates containing half-strength MS (1/2 MS) medium supplemented with 1% sucrose and 0.8% agar at 22 °C under long-day conditions. To perform the ABA sensitivity test, 5-day-old plants were transferred to plates containing 1/2 MS medium supplemented with 1% sucrose, 1.2% agar, and ABA (0, 50, or 100 μM for *35Spro::TCP13OX* and control plants; 0, 5, or 10 μM for *tcp13* and WT plants). After 7 days of incubation, the root elongation length of 7 plants per experiment was measured.

### Osmotic stress sensitivity test of transgenic and mutant plants

The osmotic stress sensitivity test was performed using the *35Spro::TCP13OX* and control plants grown in plates containing 1/2 MS medium supplemented with 1% sucrose, 30 mg/L kanamycin, and 0.8% agar or *tcp13* mutant, *tcp13tcp15tcp17* triple mutant and WT (Col-0) plants grown on1/2 MS medium supplemented with 1% sucrose and 0.8% agar at 22 °C under long-day conditions. Plates were overlaid with a nylon mesh (Prosep; pore size = 20 μm) to prevent roots from growing into the medium in a plate (diameter = 150 mm). At 9 days after sowing (DAS), when the third leaf was fully expanded, the nylon mesh was gently lifted using forceps, and seedlings were transferred to plates containing 1/2 MS medium (control) or 1/2 MS medium supplemented with 25 mM mannitol (Sigma-Aldrich). After 13 days of incubation, the leaf area of five leaves per line was measured using ImageJ.

### Dehydration tolerance test

The dehydration tolerance test was performed using the *35Spro::TCP13OX* and control plants grown in soil at 22 °C under long-day conditions. One pot contained 5 plants of each line. 3 pots of *35Spro::TCP13OXa* and *b*, and 6 pots of control plants were transferred to empty tray and exposed dehydration stress by withholding watering for 14 days in 45–60 relative humidity. After dehydration treatment, plants were grown for 7 days with well-water conditions and evaluated the survival rate of plants. We performed dehydration tolerance test three times. Total of 90 plants of control, 45 plants of *35Spro::TCP13OXa* and *b* were tested.

### Transcriptome analysis using an oligo DNA array

The Agilent Arabidopsis 4 Oligo Microarray (Agilent Technologies) containing 21,500 probes was used to identify genes downstream of *TCP13*. Total RNA was isolated from 2-week-old *TCP13pro::TCP13SRDX* whole plants using extraction buffer (0.2 M Tris-HCl [pH 9.0], 0.4 M LiCl, 25 mM EDTA, and 1% SDS). Total RNA of 10 plants of each transgenic line was pooled. This procedure was repeated to produce two biologically independent RNA pools per transgenic line. The Cy3- and Cy5-labeled cRNA of each transgenic line and control sample was hybridized to the microarray. Additionally, the color swapping experiment was performed as a technical replicate. After hybridization, the microarray slides were scanned (scanner model G2505C; scan control software version A.8.5.1; Agilent Tech, Inc., Santa Clara, CA, USA), and data were analyzed using the Feature Extraction software (version 10.10.1.1; Agilent). Raw data were analyzed using the GeneSpring GX software (version 12.0; Agilent). Expression log ratios and Benjamini–Hochberg FDR *p*-values were calculated using GeneSpring GX. The microarray design and data were deposited at ArrayExpress (Accession Number E-MTAB-9336).

### Gene expression analysis by qRT-PCR

Gene expression was analyzed by qRT-PCR as described previously (Urano et al. 2017). The AGI code- and gene-specific primers were designed based on the sequence of a single exon using Primer Express 2.0 (Applied Biosystems, Foster City, CA, USA) (Methods S1). All qRT-PCR reactions were performed in three technical replicates, and mRNA levels of genes were normalized relative to the constitutive control, *At2g32170*.

### Transactivation analysis of the *AHG3*, *Gols2*,* IAA5*, and *LBD1* promoter

Protoplasts were isolated from peeled rosette leaves of 3–4-week-old WT and *tcp13* mutant Arabidopsis plants using the Tape Arabidopsis Sandwich method (Wu et al. 2009), as described previously (Sakamoto et al. 2016; Yoshida et al. 2013). The reporter construct containing the firefly (*Photinus pyralis*) *LUC* gene driven by the *AHG3*, *Gols2*, *IAA5*, or *LBD1* promoter was co-transfected into the WT and *tcp13* mutant protoplasts using the PEG transfection method, as described previously (Sakamoto et al. 2016; Yoshida et al. 2013), along with a construct containing the modified *Renilla reniformis* luciferase gene driven by the CaMV *35S* promoter (*phRLHSP*; internal control). The transfected protoplasts were incubated at 22 °C in the dark in medium supplemented with or without 5 μM ABA or 1 μM IAA for 16–18 h. The dual-LUC assay was carried out using the Pikka Gene Dual Assay Kit (Toyo Ink, Inc., Tokyo, Japan). The reporter activity was normalized with the activity of the *Renilla* luciferase gene.

## Results

### *TCP13* shows dehydration-inducible expression

Water deficit stress induces morphological changes in plants to ensure survival. To identify key factors that control these morphological changes under dehydration stress, we analyzed the expression patterns of Arabidopsis *TCP* genes in leaves under dehydration stress using our previously published microarray data (Urano et al. 2009). Of the 24 Arabidopsis *TCP* genes on the Agilent oligo array, 11 were expressed (Fig. S1). This microarray analysis showed that only *TCP13* was upregulated under dehydration stress, whereas other *TCPs* were downregulated. The up-regulation of *TCP13* under dehydration stress is also supported by the Arabidopsis RNA-seq Database (http://ipf.sustech.edu.cn/pub/athrna/; Zhang et al. 2020) (Table S1).

**Fig. 1:**
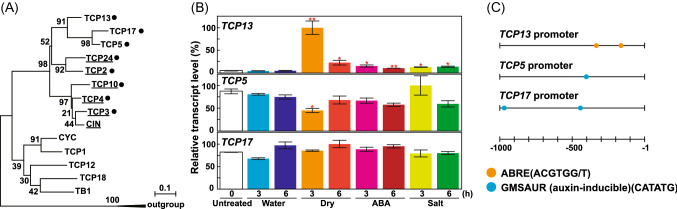
Expression pattern of *TCP13* in wild-type (WT; Col-0) Arabidopsis plants under different abiotic stresses. **a** Phylogenetic analysis of Arabidopsis class II TCP proteins and representative members of *Antirrhinum majus* CINCINNATA (CIN) and CYCLOIDEA (CYC) (Luo et al*.* 1996) families. The phylogenetic tree was constructed using the neighbor-joining method with the MEGA X software (Kumar et al. 2018). Rice (*Oryza sativa*) class I TCPs, PROLIFERATING CELL FACTOR1 (PCF1), and PCF2 (Kosugi and Ohashi 2002) were used as an outgroup. The underlined names represent proteins containing the miR319A/JAW target sequence (Palatnik et al. 2003). Black dots indicate proteins involved in leaf differentiation (Koyama et al. 2007, 2010). **b** Quantitative real-time PCR (qRT-PCR) analysis of the relative transcript levels of *TCP13, TCP5*, and *TCP17*. Plants were treated with water (control), dehydration stress, 10 μM abscisic acid (ABA), or salt stress (175 mM NaCl), and gene expression was examined at 3 and 6 h post-treatment. Data represent mean ± standard deviation (SD; *n* = 3). Asterisks indicate significant differences (*P < 0.05, **P < 0.01; one-way ANOVA with Welch’s *t* test). **c** Schematic showing the structure of the 1000 bp promoter of *TCP13*, *TCP5*, and *TCP17*. Red and blue circles indicate the ABA-responsive element (ABRE)-related sequences (ACGTGG/T) (Busk and Pages 1998; Yamaguchi-Shinozaki and Shinozaki 2006) and GMSAUR (CATATG) motif found in the promoter of the soybean *SAUR15A* gene (Xu et al. 1997), respectively

Amino acid sequence alignment of Arabidopsis class II TCPs suggests that TCP13 is relatively similar to TCP5 and TCP17 (Fig. 1a). *TCP5*, *TCP13*, and *TCP17* do not contain the *miR319A/JAW* target sequence (Palatnik et al. 2003), and all three TCPs have been previously shown to be involved in leaf differentiation, which is a common function of CIN-like TCPs (Koyama et al. 2007, 2010). Figure 1b shows the results of quantitative real-time PCR (qRT-PCR) analysis of *TCP5*, *TCP13*, and *TCP17* in Arabidopsis plants exposed to ABA, high salt, or dehydration stress for 3 or 6 h. The expression of *TCP13* was highly upregulated during the early phase of dehydration stress, and slightly upregulated by exogenous ABA and high salinity. In contrast, the expression of *TCP5* decreased during the early phase of dehydration. Expression of *TCP17* was not affected by any of the three abiotic stresses (Fig. 1B).

To investigate why the response of *TCP13* to abiotic stress was different from that of *TCP5* and *TCP17*, we analyzed the promoter sequences of all three genes. The *TCP13* promoter contained two typical ABRE motifs (ACGTGG) (Fig. 1c). By contrast, *TCP5* and *TCP17* promoters lacked the ABRE motif but contained an auxin-responsive element, named GMSAUR (CATATG), which was found in the promoter of an auxin-responsive gene, *SAUR15A*, of soybean (*Glycine max*) (Xu et al. 1997). These results suggest that TCP13 might perform a unique function under dehydration stress via ABA signaling. In addition, amino acid sequence alignment of class II TCPs of selected vascular land plants such as Arabidopsis, *Brassica napus*, soybean, alfalfa (*Medicago truncatula*), rice (*Oryza sativa*), maize (*Zea mays*), and a bryophyte (*Physcomitrella patens*) was shown in Fig. S2.

### Tissue-specific expression of *TCP13*, and subcellular localization of TCP13 protein

**Fig. 2:**
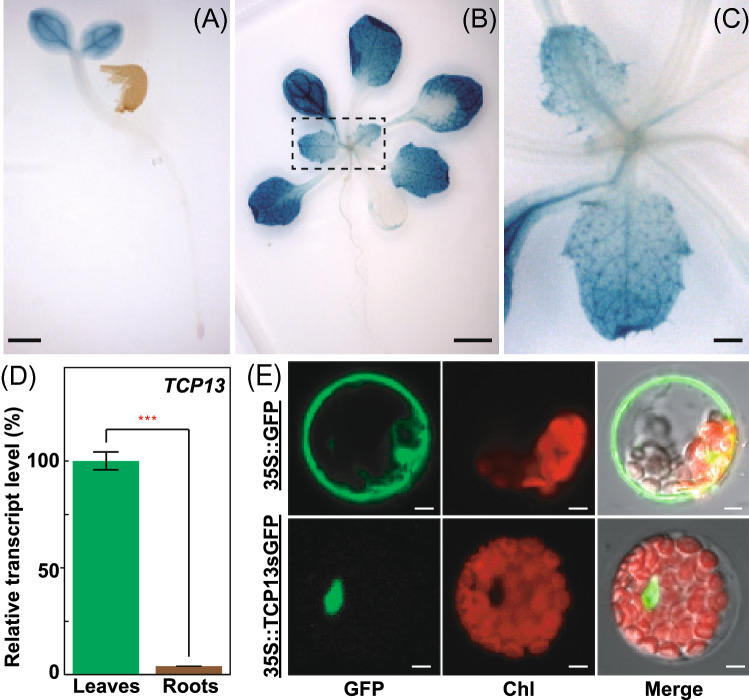
Expression pattern of *TCP13* in Arabidopsis plants at different developmental stages, and subcellular localization of the TCP13 protein. **a**–**c** Histochemical staining showing β-glucuronidase (GUS) reporter activity (blue) under the control of the *TCP13* promoter in seedlings (**a**), leaves (**b**), and in an enlarged view of the true leaves (**c**). Scale bars = 1 mm (**a** and **c**); 5 mm (**b**). **d** Analysis of the relative transcript level of *TCP13* in leaves and roots by qRT-PCR. Transcript levels of genes were normalized relative to the constitutive control, *At2g32170* (Czechowski et al. 2005). Data represent mean ± SD (*n* = 3). Asterisks indicate significant differences (**P < 0.001; one-way ANOVA with Welch’s *t* test). **e** Subcellular localization of TCP13-sGFP fusion protein in Arabidopsis mesophyll protoplasts. Scale bars = 5 μm

To analyze the tissue-specific expression profile of *TCP13*, we performed histochemical staining of transgenic Arabidopsis plants expressing the *β-glucuronidase* (*GUS*) gene under the control of the *TCP13* promoter (*TCP13pro::GUS*) (Fig. 2a–c), and analyzed the expression of *TCP13* in leaves and roots of wild-type (WT) plants by qRT-PCR (Fig. 2d). GUS activity was detected in the leaves, but not in the roots, of 5- and 10-day-old plants (Fig. 2a and b). GUS staining was detected in cotyledon and true leaves (Fig. 2a and b). GUS staining was specifically detected in expanding cells (upper region) but not in dividing cells (lower region) in young leaves (Fig. 2c). Additionally, qRT-PCR analysis showed that the expression level of *TCP13* was high in leaves and low in roots (Fig. 2d). Tissue-specific *TCP13* expression data obtained from the Arabidopsis EFP browser (http://bar.utoronto.ca/efp/cgi-bin/efpWeb.cgi) (Winter et al. 2007) showed that *TCP13* is expressed in mesophyll cells, but not in guard cells, of leaves (Fig. S3a), and in the endodermis and cortex of roots under salt stress (Fig. S3b).

To determine the subcellular localization of TCP13, we fused the full-length *TCP13* gene to a synthetic *green fluorescent protein* (*sGFP*) gene (*TCP13-sGFP*), and introduced the construct into Arabidopsis leaf protoplasts via polyethylene glycol (PEG)-mediated transfection. The TCP13-sGFP fusion protein was transiently expressed in the nuclei (Fig. 2e). Together, these data suggest that *TCP13* exhibits strong expression in leaves, and the encoded protein localizes to the nuclei.

### Characterization of *TCP13* overexpression (*TCP13OX*) lines and *tcp5tcp13tcp17* triple mutant plants under normal growth conditions

**Fig. 3:**
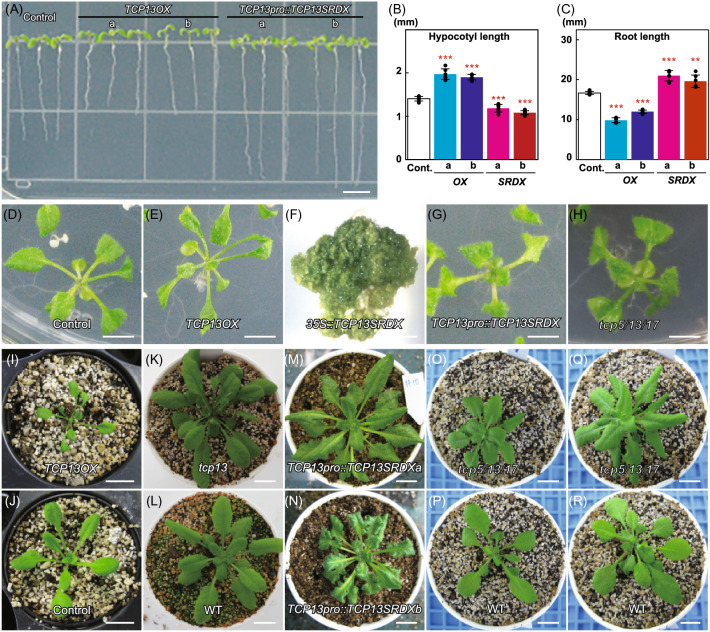
Characterization of *TCP13* overexpression (*TCP13OX*) lines and *tcp13* loss-of-function mutant plants at different developmental stages. **a** Root growth of 7-day-old *35Spro::TCP13OX* and *TCP13pro::TCP13SRDX* seedlings. Scale bars = 5 mm. **b** and **c** Hypocotyl (**b**) and root (**c**) length of 7-day-old *35Spro::TCP13OX* and *TCP13pro::TCP13SRDX* seedlings. Data represent mean ± SD (*n* = 6). An asterisk shows that the indicated mean is significantly different from the mean value of the control plant (**P < 0.01, ***P < 0.001; one-way ANOVA with Welch’s *t* test). **d**–**h** Images of 14-day-old empty vector control (**d**), *35Spro::TCP13OX* (**e**), *35Spro::TCP13SRDX* (**f**), *TCP13pro::TCP13SRDX* (**g**), and *tcp5/13/17* triple mutant (**h**) plants grown in agar. Scale bar = 5 mm (**d**, **e**, **g**, and **h**); 1 mm (**f**). **i**–**r** Images of 21-day-old *35Spro::TCP13OX* (**i**) and corresponding empty vector control (**j**) plants; 28-day-old *tcp13* mutant (**k**) and corresponding WT (**l**) plants; 28-day-old *TCP13pro::TCP13SRDX* (m and n) plants; 21-day-old *tcp5/13/17* (**o**) and corresponding WT (**p**) plants; and 28-day-old *tcp5/13/17* mutant (**q**) and corresponding WT (**r**) plants. Scale bars = 10 mm

First, we analyzed transgenic plants overexpressing *TCP13* cDNA under the control of the constitutive Cauliflower mosaic virus (CaMV) *35S* promoter (*35Spro::TCP13OX*). The *35Spro::TCP13OX* seedlings showed longer hypocotyls and shorter roots than plants transformed with the empty vector (control) (Fig. 3a–c). True leaves of *35Spro::TCP13OX* plants were narrower than those of control plants and showed downward rolling (Fig. 3d, e, i, and j). We also examined the effect of *TCP13* repression on plant morphology. The Chimeric REpressor Silencing Technology (CRES-T) is a gene silencing system, in which a TF fused to the EAR-motif repression domain (Leu-Asp-Leu-Asp-Leu-Glu-Leu-Arg-Leu-Gly-Phe-Ala;SRDX) dominantly represses the transcription of its target genes, regardless of the absence or presence of endogenous and functionally redundant TFs (Hiratsu et al. 2003). We generated transgenic plants expressing *SRDX*-fused *TCP13* cDNA (*TCP13SRDX*). Transgenic Arabidopsis plants expressing the chimeric repressor under the control of the constitutive CaMV 35S promoter (*35Spro::TCP13SRDX*) produced ectopic shoots and failed to grow when transferred to soil (Fig. 3f). Transgenic seedlings expressing the chimeric repressor under the control of the *TCP13* promoter (*TCP13pro::TCP13SRDX*) showed greater root growth (Fig. 3a and c) and wavy and serrated rosette leaves (Fig. 3g, m, and n) compared with control plants (Fig. 3d and j). Rosette leaves of *tcp13* mutant plants showed no severe phenotypic abnormality (Fig. 3k) compared with those of WT plants (Fig. 3l). By contrast, *tcp5tcp13tcp17* triple knockout mutant plants (*tcp5/13/17)* showed shorter petioles and smaller leaves (Fig. 3h and o), with slightly wavy and serrated margins (Fig. 3q), compared with the respective control (Fig. 3d) or WT plants (Fig. 3p and r), suggesting that *TCP13*, together with *TCP5* and *TCP17*, regulates leaf morphology.

We also generated transgenic Arabidopsis plants overexpressing *TCP5* and *TCP17* under the control of the constitutive CaMV *35S* promoter (*35Spro::TCP5OX* and *35Spro::TCP17OX*, respectively). Transgenic *35Spro::TCP5OX* and *35Spro::TCP17OX* seedlings showed longer hypocotyls than empty vector-transformed control plants (Fig. S4a). This phenotype of *35Spro::TCP5OX* and *35Spro::TCP17OX* seedlings was similar to that of transgenic seedlings overexpressing a mutant form of *TCP3* (*35Spro::mTCP3OX*), in which the target site of miR319/JAW was replaced with a non-target sequence (Koyama et al. 2007), and to that of previously generated transgenic seedlings overexpressing *TCP5* (*35Spro::TCP5OX*) (Han et al. 2019) and *TCP17* (*35Spro::TCP17OX*) (Han et al. 2019; Zhou et al. 2019). Furthermore, *TCP5*, *TCP13*, and *TCP17* were shown to play basic roles in promoting thermo-responsive hypocotyl growth by positively regulating PIF4 activity (Han et al. 2019; Zhou et al. 2019). In contrast to *35Spro::TCP13OX* plants, the *35Spro::TCP5OX* and *35Spro::TCP17OX* plants showed no change in root growth in comparison with control plants (Fig. S4b). These results suggest that inhibition of root growth is a unique function of *TCP13* among *CIN-like TCP* genes*.*

### TCP13 inhibits leaf and root growth in response to ABA and osmotic stress, and is involved in tolerance to dehydration stress

**Fig. 4:**
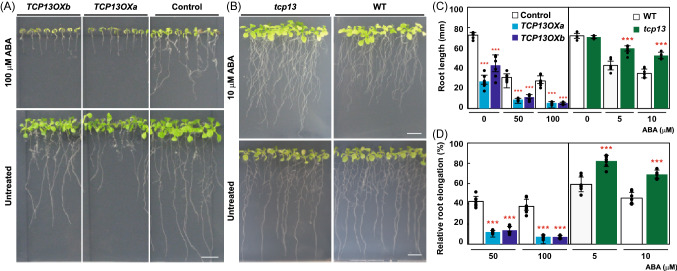
Characterization of *TCP13OX* and *tcp13* mutant plants in the ABA treatment. Five-day-old seedlings were transferred to control and ABA-containing medium and grown for 7 days and root length was measured. Root elongation under the ABA treatment was compared with that under the control treatment, and the rate of root elongation was calculated. **a** and **b** Images of the root growth of *35Spro::TCP13OX* transgenic and control plants (**a**) and *tcp1*3 mutant and WT plants (**b**) in the ABA treatment. Scale bars = 10 mm. **c** The root length of *35Spro::TCP13OX* transgenic and control plants (left panel) and *tcp1*3 mutant and WT plants (right panel) in the ABA treatment. **d** Relative level of root elongation of *35Spro::TCP13OX* transgenic and control plants (left panel) and *tcp1*3 mutant and WT plants (right panel) in the ABA treatment. In each transgenic or mutant plants, average of root length in the control condition was set to 100. Data represent mean ± SD (*n* = 7). An asterisk shows that the indicated mean is significantly different from the mean value of the wild-type plant under the corresponding condition (***P < 0.001, one-way ANOVA with Welch’s *t* test)

The *TCP13* gene is significantly induced by dehydration stress and slightly induced by high salinity and ABA treatments (Fig. 1b). To analyze the function of *TCP13* in ABA response, we examined the response of the *35Spro::TCP13OX* transgenic seedling and *tcp13* knockout mutant seedlings to the ABA treatment (Fig. 4). To carry out the ABA sensitivity test, the *35Spro::TCP13OX* and *tcp13* plants grown on agar plates for 5 days were transferred to plates containing agar supplemented with or without ABA. After 7 days, the root length of these seedlings was measured (Fig. 4c), and the root growth in the presence of ABA was compared with that in the absence of ABA (Fig. 4d). The *35Spro::TCP13OX* plants showed reduced root growth compared with control plants in the absence and presence of ABA treatment (Fig. 4a and c). The enhanced root growth retardation relative to the control plants was detected in the *35Spro::TCP13OX* plants under ABA treatment (Fig. 4d). In contrast, the WT and *tcp13* mutant plants showed similar growth in the absence of ABA, whereas the *tcp13* mutant plants showed greater root elongation than WT plants in response to ABA (Fig. 4b, c and d). These results suggest that TCP13 negatively regulates root growth in response to the ABA treatment.

**Fig. 5:**
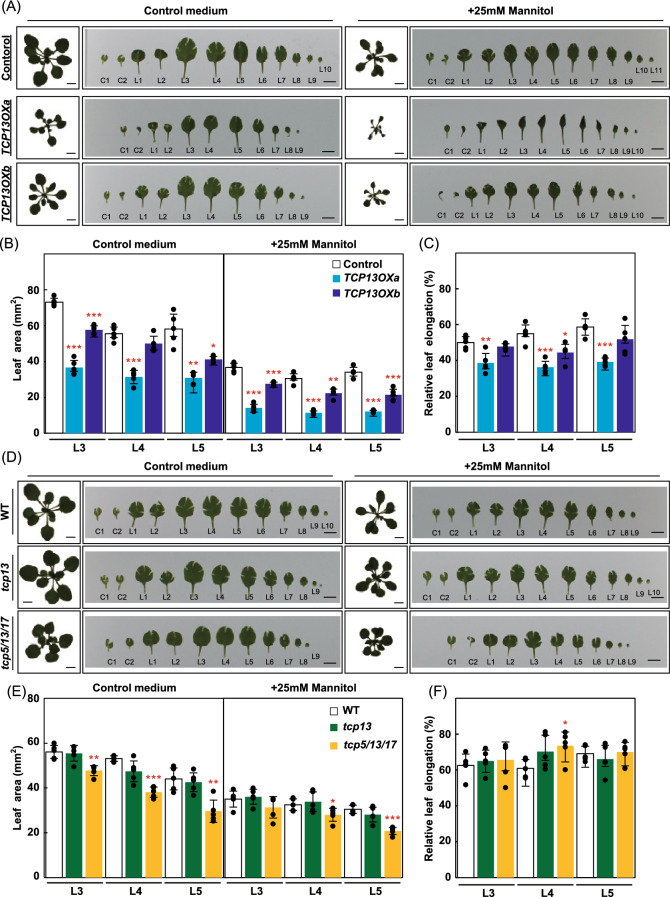
Characterization of *35Spro::TCP13OX*, *tcp13* and *tcp5/13/17* mutant plants under osmotic stress conditions. Nine-day-old *35Spro::TCP13OX* and control plants, and *tcp13* mutant, *tcp5/13/17* mutant and WT plants were transferred to 1/2 MS medium containing 25 mM mannitol for 13 days. The leaf area of third (L3), fourth (L4), and fifth (L5) leaves was measured. Leaf growth under the mannitol treatment was compared with that under the control treatment, and the rate of leaf growth was calculated. **a** and **d** Images of cotyledon (C) and true leaves (L) of *35Spro::TCP13OX* and control plants (**a**) and *tcp13* mutant, *tcp5/13/17* mutant and WT plants (**d**) exposed to control and mannitol treatments. Scale bars = 5 mm. **b** and **e** The leaf area in *35Spro::TCP13OX* transgenic and control plants (**b**) and *tcp13* mutant, *tcp5/13/17* mutant and WT plants (**e**) treated with control and mannitol medium. **c** and **f** Relative level of leaf growth in *35Spro::TCP13OX* transgenic and control plants (**c**) and *tcp13* mutant, *tcp5/13/17* mutant and WT plants (**f**) treated with mannitol. In each transgenic or mutant plants, average of leaf area in the control condition was set to 100. Data represent mean ± SD (*n* = 5). An asterisk shows that the indicated mean is significantly different from the mean value of the wild-type plant under the corresponding condition (*P < 0.05, **P < 0.01, ***P < 0.001; one-way ANOVA with Welch’s *t* test)

To analyze the effect of *TCP13* on plant response to osmotic stress, we examined changes in the leaf growth of the *35Spro::TCP13OX*, *tcp13* and *tcp5/13/17* knockout mutant plants on agar plates under mannitol stress (Fig. 5). Nine-day-old plants were transferred to plates containing half-strength Murashige and Skoog (1/2 MS) medium or 1/2 MS medium supplemented with 25 mM mannitol, which has been shown to restrict leaf growth by approximately 50% (Skirycz et al. 2011). After 13 days, each rosette leaf was separated (Fig. 5a and d), and size of third (L3), fourth (L4) and fifth (L5) leaf in the mannitol treatment was compared with that in the control treatment (Fig. 5b, c, e, and f). The *35Spro::TCP13OX* plants showed growth retardation with smaller leaves compared with the control plants in control and mannitol conditions (Fig. 5 a and b). Especially, leaves of *35Spro::TCP13OXa* plants in the mannitol treatment showed severe rolling from the mid-point to the tip (Fig. 5a). The enhanced leaf growth retardation relative to the control plants was detected in L3-L5 of the *35Spro::TCP13OXa* and L4 of the *35Spro::TCP13OXb* plants under mannitol treatment (Fig. 5c). These results indicate that *35Spro::TCP13OX* leaves are hypersensitive to osmotic stress. On the other hand, the *tcp13* mutant plants treated with mannitol showed no significant changes in leaf growth compared with control plants (Fig. 5d–f). *tcp5/13/17* triple mutant plants showed growth retardation with smaller leaves compared with the control plants in control (L3-5) and mannitol (L4 and L5) conditions (Fig. 5e). The reduced leaf growth retardation relative to the WT plants was detected in L4 of the *tcp5/13/17* mutant plants under mannitol treatment (Fig. 5f), indicating that *tcp5/13/17* showed insensitive response to osmotic stress. *TCP13* probably exhibit functional redundancies between *TCP5* and *17* for leaf growth regulation under osmotic stress.

**Fig. 6:**
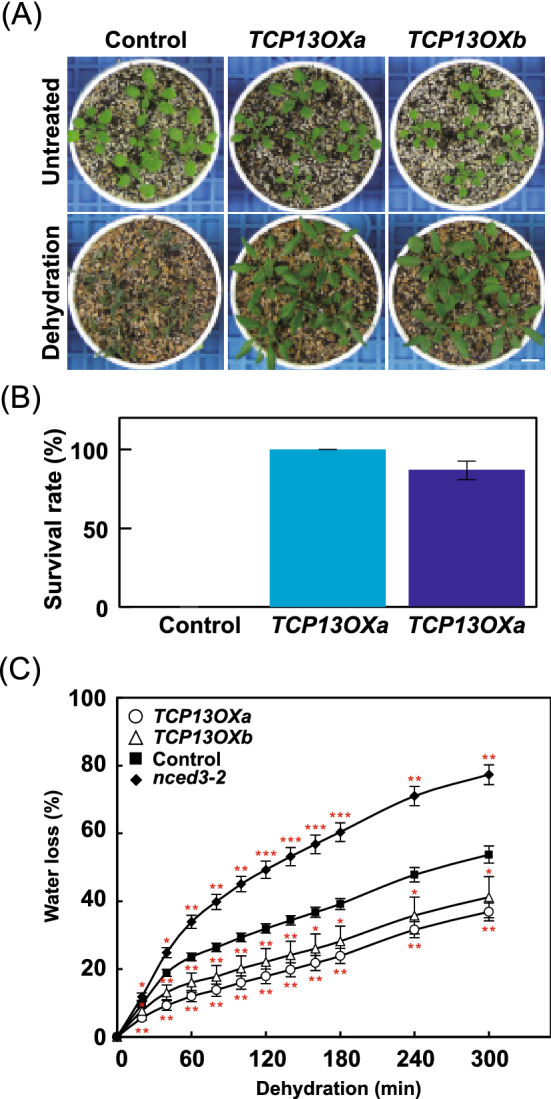
Characterization of *35Spro::TCP13OX* plants under severe dehydration stress. *35Spro::TCP13OX* and control plants were grown in soil for 14 days with well-water conditions. One pot contained 5 plants of each line. 3 pots of *35Spro::TCP13OX* 35S*a* and *b*, and 6 pots of control plants were transferred to empty tray and exposed dehydration stress by withholding watering for 14 days. After dehydration treatment, plants were grown for 7 days with well-water conditions and evaluated the survival rate of plants. The dehydration tolerance test was performed three times. **a** Images of plants before dehydration stress (Untreated) and after dehydration (Dehydration). Scale bars = 10 mm. **b** The survival rate of plants after dehydration. The error bars indicate the SD from three replicates. *n* = 90 (WT), n = 45 (*TCP13OXa*) and n = 45 (*TCP13OXb*), total. **c** Water loss assay of detached leaves of 5-week-old *35Spro::TCP13OX* 35S and control plants. The *nced3-2* plants represent a negative control. Data represent mean ± SD (*n* = 4). An asterisk shows that the indicated mean is significantly different from the mean value of the control plant under the corresponding condition (*P < 0.05, **P < 0.01, ***P < 0.001, one-way ANOVA with Welch’s *t* test)

We then examined the response of *35Spro::TCP13OX* plants to severe dehydration stress. Watering of *35Spro::TCP13OX* plants was withheld for 14 days, and the survival rate of plants was calculated at 7 days after rewatering. The *35Spro::TCP13OX* plants showed greater dehydration stress tolerance, as evident from the reduced downward leaf rolling phenotype and higher survival rate, than control plants (Fig. 6a and b). This result was consistent with a lower reduction in the water content of *35Spro::TCP13OX* plants, based on the measurement of the weight of detached leaves, than in the water content of control plants (Fig. 6c). On the other hand, detached leaves of *nced3-2* mutant plants (negative control), carrying a knockout mutation in a key gene responsible for dehydration-inducible ABA accumulation, showed a greater reduction in the water content than control plants (Fig. 6c). These results suggest that leaves of *35Spro::TCP13OX* plants show less water loss than control plants under dehydration stress.

### Transcriptome analysis of *TCP13pro::TCP13SRDX* plants

**Fig. 7:**
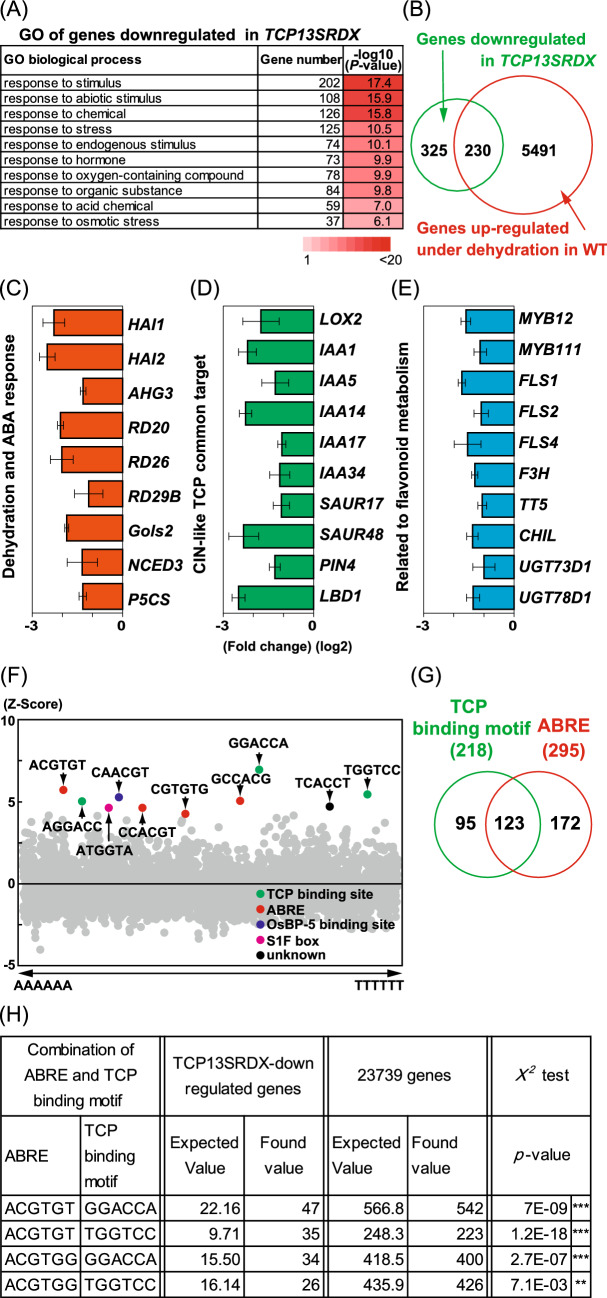
Transcriptome analysis of genes downregulated in *TCP13pro::TCP13SRDX* plants. **a** GO enrichment analyses of downregulated in *TCP13pro::TCP13SRDX* plants. **b** Venn diagram showing the overlap between genes downregulated in *TCP13pro::TCP13SRDX* plants and those upregulated by dehydration stress (Urano et al. 2017). **c**–**e** Relative transcript levels of downregulated genes including ABA- and dehydration-inducible genes (**c**), *CIN-like TCP* common downstream genes involved in auxin signaling (**d**), and flavonoid biosynthesis genes (**e**) in *TCP13pro::TCP13SRDX* plants. Data represent mean ± SD (*n* = 4). **f** Enrichment of hexamer motifs in the promoter regions (~ 1000 bp) of downstream candidate genes of *TCP13*. Hexamer motifs in the promoter regions of the top 100 downregulated genes in *TCP13pro::TCP13SRDX* plants were analyzed as described previously (Maruyama et al. 2012). **g** Comparison of TCP binding and ABRE motifs identified in the promoter regions of downregulated genes in *TCP13pro::TCP13SRDX* plants. **h** Comparison of the 1000 bp promoter regions of *TCP13SRDX*-downregulated genes with those of 23,739 Arabidopsis genes used previously for motif analyses (Maruyama et al. 2012) by χ^2^ tests. ACGTGT and ACGTGG were used as the ABRE sequences, and GGACCA and TGGTCC were used as the TCP binding motifs. Significant differences between the observed and expected values of the frequency of overlap between the ABRE and TCP binding motifs (**P < 0.01, ***P < 0.001)

To investigate the transcriptional regulation of *TCP13*, we performed microarray analysis of whole plants expressing the *TCP13pro::TCP13SRDX* and focused on genes downregulated in *TCP13pro::TCP13SRDX* plants (Fig. 7)*.* The results showed that 555 genes were downregulated in *TCP13pro::TCP13SRDX* plants (fold-change [FC] < 0.5; *p* < 0.05; false discovery rate [FDR] < 0.0277) (Table S2) compared with control plants.

Next, we performed Gene Ontology (GO) enrichment analysis (PANTHER Classification System, https://www.arabidopsis.org/tools/go_term_enrichment.jsp) of these 555 genes to determine their potential functions. A total of 48 GO terms were significantly enriched (*p* < 0.05) (Table S3), and the top 10 categories are shown in Fig. 7a. Among these GO terms, “response to stimulus” was the most highly enriched GO term, followed by “response to abiotic stimulus” and “response to chemical”. Then, we compared the 555 genes downregulated in *TCP13pro::TCP13SRDX* plants with dehydration-inducible genes reported previously (Urano et al. 2017). The results showed that 230 out of 555 genes were upregulated under dehydration stress in the study of Urano et al. (2017) (FC < 2; *p* < 0.05) (Fig. 7b). These results suggest that numerous downstream candidates of TCP13 are involved in dehydration stress response.

We further analyzed the expression of dehydration stress-responsive and ABA-regulated genes downregulated in *TCP13pro::TCP13SRDX* plants in our microarray results (Fig. 7c). These genes included *Highly ABA-induced Protein Phosphatase 2C 1* (*HAI1*), *HAI2*, *ABA-hypersensitive Germination3* (*AHG3*), *Response to Desiccation 20* (*RD20*), *RD26*, and *RD29B*, and key genes involved in raffinose, proline, and ABA metabolism such as *Galactinol Synthase2* (*GolS2*), *delta-1-pyrroline-5-carboxylate synthase1* (*P5CS1*), and *9-cis-epoxycarotenoid dioxygenase3* (*NCED3*) (Fig. 7c). These genes were not identified as downstream candidates of CIN-like TCPs in previous studies (Koyama et al. 2007, 2010, 2017; Schommer et al. 2008) but were newly identified as downstream candidates of only the dehydration-inducible TCP13. In addition, we also found genes related to the common targets of CIN-like TCP (Koyama et al. 2007, 2010, 2017; Schommer et al. 2008) as being potential downstream candidates of TCP13 in our microarray results (Fig. 7d, e). These included related to JA metabolic gene and auxin-regulated genes such as *LOX2*, *IAA1*, *IAA5*, *IAA14*, *IAA17*, *IAA34*, *SAUR17*, *SAUR34*, *PIN4*, and *LBD1* (Fig. 7d), and related to flavonoid metabolic genes such as *MYB12*, *MYB111*, *Flavonol Synthase1* (*FLS1*), *FLS2*, *FLS4*, *Chalcone Isomerase* (*CHI/TT5*), *CHI-like* (*CHIL*), *flavone 3-hydroxylase* (*F3H/TT6*), *UDP-glucosyl transferase 73B2* (*UGT73B2*), and *UGT78D1* (Fig. 7e). We also analyzed the expression of ABA-regulated genes such as *AHG3*, *RD20*, *Gols2*, and *NCED3*, and auxin-regulated genes, such as *IAA5* and *LBD1*, in *35Spro*::*TCP13-OX* plants. Among these genes, *Gols2*, *NCED3*, *IAA5*, and *LBD1* were upregulated in *35Spro*::*TCP13-OX* plants compared with WT plants (Figure S8). These results revealed that expression of *TCP13SRDX* triggers changes in the expression patterns of downstream genes regulated specifically by TCP13 as well as those regulated by various CIN-like TCPs.

Furthermore, to identify common motifs present in the promoters of the top 100 most downregulated genes in *TCP13pro::TCP13SRDX* plants, we searched the frequency of hexamer motifs in 1000 bp promoter regions (Table S4), as described previously (Maruyama et al. 2012). Among the top 10 motifs identified in gene promoters, three TCP binding motifs-related sequences (GGACCA) (Schommer et al. 2008) and four ABRE-related sequences (ACGTGG/T) (Busk and Pages 1998; Yamaguchi-Shinozaki and Shinozaki 2006) were found (Fig. 7f). Among all 555 downregulated genes, 218 genes contained the TCP binding motifs in their promoter regions, and 123 genes contained both TCP binding and ABRE motifs (Fig. 7g). The repressive activity of *TCP13* induced by the SRDX motif accurately downregulated TCP-regulated downstream genes, most of which were also responsive to ABA signaling or dehydration stress. Although many downstream candidate genes of TCP13 contained the ABRE motifs in their promoters, TCP13 probably does not directly bind to these motifs (Fig. S6). To statistically clarify the overlap between ABRE sequences (ACGTGG and ACGTGT) and TCP binding motifs (GGACCA and TGGTCC) in the promoter regions of genes downregulated by *TCP13SRDX*, we performed χ^2^ tests to compare the 1000 bp promoter region of *TCP13SRDX*-downregulated genes with that of 23,739 Arabidopsis genes previously used for motif analyses (Maruyama et al. 2012) (Fig. 7h). Significant differences were detected between the observed and expected values of the frequency of overlap between the ABRE and TCP binding motifs in the 1000 bp promoter regions of 218 *TCP13SRDX*-down-regulated genes. Combinations of typical ABRE sequences (ACGTGG and ACGTGT) with the TCP binding motifs (GGACCA or TGGTCC) were frequently found in the promoter regions of *TCP13SRDX*-downregulated genes. These results show that TCP13-target genes harbor both TCP binding and ABRE motifs in their promoter regions.

### Genes downregulated in* tcp13* mutant plants under dehydration stress

**Fig. 8:**
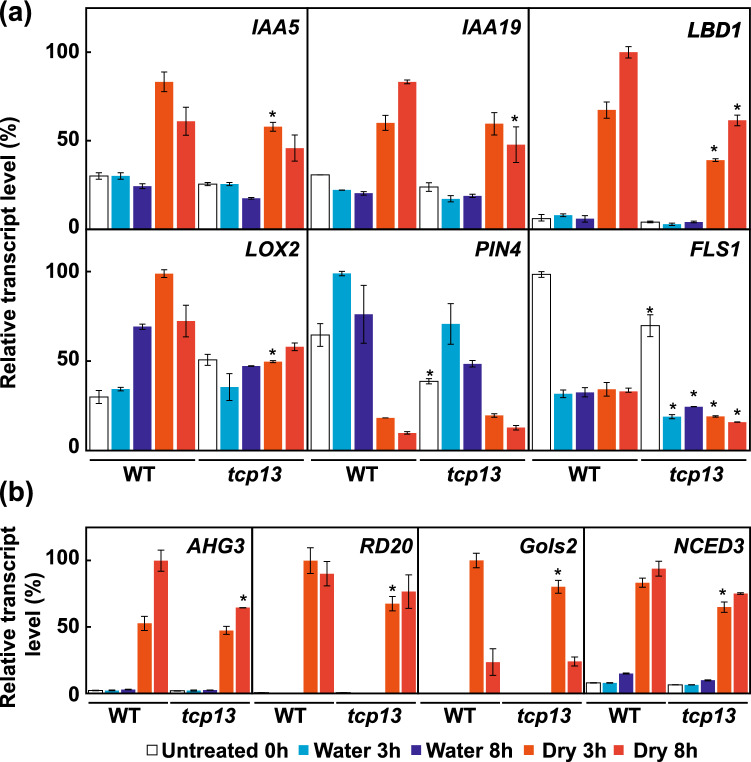
Expression analysis of *TCP13* downstream genes in *tcp13* mutant plants under dehydration stress. **a** and **b** Expression of common downstream target genes of CIN-like TCPs (**a**), and expression of ABA-inducible genes (**b**) analyzed by qRT-PCR. Transcript levels of genes were normalized relative to the constitutive control, *At2g32170* (Czechowski et al. 2005). In each experiment, the maximum gene transcript level was set to 100. Data represent mean ± SD (*n* = 3 technical replicates). An asterisk shows that the indicated mean is significantly different from the mean value of the wild-type plant under the corresponding condition (*P < 0.05, one-way ANOVA with Welch’s *t* test)

To investigate the transcriptional regulation by TCP13 under dehydration stress conditions, the expression of genes downregulated in *TCP13pro::TCP13SRDX* plants (determined by microarray) was examined in *tcp13* mutant plants under dehydration stress by qRT-PCR (Fig. 8). First, we analyzed the expression of common downstream targets of CIN-like TCPs by qRT-PCR. *IAA5* and closely related genes, *IAA19*, *LBD1*, and *LOX2*, showing reduced expression in *tcp13* mutant plants (Fig. 8a). Compared with the WT, the expression of *IAA5* and *LOX2* was slightly reduced at 3 h after dehydration stress, while that of *IAA19* induction was reduced at 8 h in *tcp13* mutant plants. The expression of *LBD1* was reduced at both 3 and 8 h after dehydration stress in *tcp13* mutant plants compared with the WT. By contrast, *PIN4* and *FLS1* expression levels were reduced in *tcp13* mutant plants compared with the WT under normal conditions.

We found that dehydration-inducible expression of selected ABA-regulated genes was also reduced in *tcp13* mutant plants compared with WT plants (Fig. 8b). The expression of *RD20*, *NCED3*, and *GolS2* genes was slightly reduced at 3 h after the dehydration treatment, while that of *AHG3* was reduced at 8 h after dehydration stress in *tcp13* mutant plants. We also compared the expression of *AHG3*, *RD20*, *Gols2*, and *NCED3* between ABA-treated WT and ABA-treated *tcp13* mutant plants (Figure S7). These genes were upregulated in WT plants, but those upregulations were reduced in *tcp13* mutant plants after the ABA treatment. Knockout of *TCP13* slightly reduced the response of ABA- and dehydration-inducible genes under dehydration stress. These results suggest that TCP13 is involved in the regulation of not only common target genes of CIN-like TCPs but also ABA- and dehydration-inducible genes.

### Transcriptional regulation of ABA- and auxin-regulated genes by TCP13

**Fig. 9:**
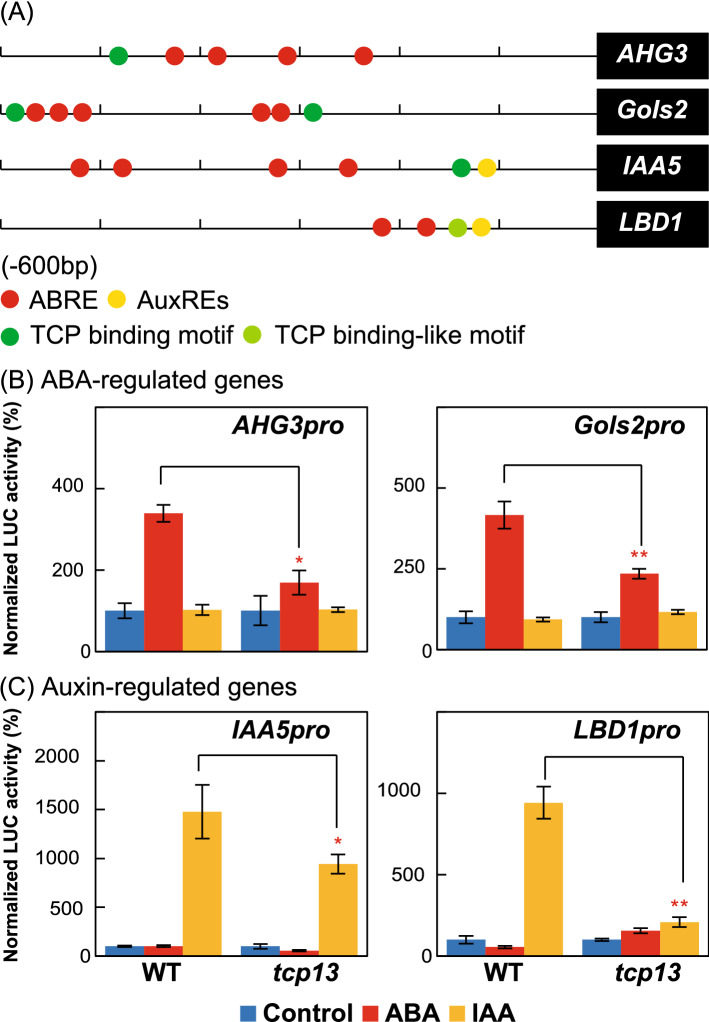
Protoplast transient assays of ABA- and auxin-regulated genes in *tcp13* mutant plants. **a** Existence of the ABREs (ACGTGT, ACGTGG, ACACGT, and CCACGT), AuxREs (TGTCTC and GAGACA), and TCP binding motifs (GGACCA, GGTCCT, TGGTCC, and AGGACC) in *AHG3*, *Gols2*, *IAA5*, and *LBD1* promoters. The *LBD1* promoter also contained the TCP binding motif like (TGGTCA). **b** and **c** Protoplast transient assays for the activation analysis of ABA-regulated genes (**b**) and auxin-regulated genes (**c**) in WT and *tcp13* mutant protoplasts in response to 5 μM ABA or 1 μM IAA. In each experiment, relative luciferase (LUC) activity in the control condition was set to 100. Data represent mean ± SD (*n* = 3 technical replicates). An asterisk shows that the indicated mean is significantly different from the mean value of the wild-type plant under the corresponding condition (*P < 0.05, **P < 0.01, one-way ANOVA with Welch’s *t* test)

The results of qRT-PCR analysis showed that selected ABA-regulated genes (such as *AHG3* and *Gols2*) and common target genes of CIN-like TCPs (such as *IAA5* and *LBD1*) were downregulated in *tcp13* mutant plants after dehydration stress (Fig. 8). The IAA/AUX proteins are auxin-sensitive repressors (Weijers and Wagner 2016). To clarify the effect of ABA- and auxin-regulated genes by TCP13, we selected *AHG3* and *Gols2* as the TCP-regulated ABA signaling genes and *IAA5* and *LBD1* as the TCP-regulated auxin signaling genes for reporter assay (Fig. 9). We investigated the activity of *AHG3, Gols2*, *IAA5* and *LBD1* gene promoter fused to the *luciferase* (*LUC*) reporter in mesophyll protoplasts of WT and *tcp13* in response to the ABA and IAA treatments (Fig. 9). Figure 9a showed the existence of three DNA binding motifs of ABRE, auxin-response elements (AuxREs) (Ulmasov et al. 1995) and TCP binding motif in the promoter regions of *AHG3, Gols2*, *IAA5* and *LBD1. AHG3* and *Gols2* harbor both ABRE and TCP binding motifs, whereas those of *IAA5* and *LBD1* contain three types of motifs including the ABRE, AuxRE, and TCP binding motif or TCP binding motif like. The *AHG3* and *Gols2* promoters-LUC activities increased in WT plants and those activities were reduced in the *tcp13* mutant plants after the ABA treatment (Fig. 9b). However, the activities of these promoters-LUC were not affected by the IAA treatment, indicating that *TCP13* is required for the activation of *AHG3* and *Gols2* genes during ABA signaling. *IAA5* and *LBD1* promoter-LUC activities increased in WT plants and those activity were reduced in *tcp13* mutant plants after the IAA treatment (Fig. 9c). However, the activities of these promoters-LUC were not affected by the ABA treatment, indicating that *TCP13* is required to activates *IAA5* and *LBD1* during auxin signaling.

## Discussion

### A possible role of TCP13 in plant growth regulation in response to dehydration stress

Leaves often change their architecture and growth to cope with environmental fluctuations, such as limited water availability (Hsiao et al. 1984; Zhang et al. 2018). Leaf rolling is a spontaneous response of plants to dehydration stress. Water deficit causes downward rolling of leaves that prevents water loss and modulates the speed of plant growth in Arabidopsis (Fujita et al. 2018). However, factors regulating these morphological changes under water limiting conditions have not yet been elucidated. Identification of the genetic components that modulate leaf architecture and growth may lead to useful strategies to enhance crop yield under stress conditions.

In this study, we demonstrated that the CIN-like TCP TF, TCP13, plays a pivotal role in the regulation of plant growth under dehydration stress conditions. Among the *CIN-like TCP* gene family members, *TCP13* is significantly upregulated by dehydration stress (Fig. 1). The *TCP13* promoter contains two ABRE motifs. The *TCP13OX* transgenic Arabidopsis plants produced narrow leaves, with the downward rolling phenotype, compared with control plants, and these leaf phenotypes were drastically enhanced in response to the mannitol treatment and dehydration stress (Figs. 3, 5 and 6). Additionally, *TCP13OX* transgenic plants showed higher tolerance and reduced water loss under dehydration stress (Fig. 6). These results led us to speculate that TCP13 modulates the leaf growth to cope with dehydration stress conditions. The leaf growth retardation of the *tcp5/13/17* triple mutants were slightly reduced in response to the mannitol treatment, whereas the *tcp13* mutant plants showed no specific leaf phenotype under osmotic stress conditions (Fig. 5). These observations indicate that TCP13 probably contributes to the leaf growth together with TCP5 and TCP17 under stress conditions.

Genome-wide expression and qRT-PCR analyses revealed that *IAA1, 5, 14, 17*, and *34* were repressed in *TCP13pro::TCP13SRDX* plants (Fig. 7), and dehydration-inducible expression of *IAA5* and *19* was reduced in *tcp13* mutant plants under dehydration stress (Fig. 8). The IAA/AUX proteins are auxin-sensitive repressors that mediate diverse physiological and developmental processes in plants (Weijers and Wagner 2016). The *incurvata6* (*icu6*) semi-dominant allele of the *AUXIN RESISTANT3* (*AXR3*)/*IAA17* gene increases auxin response and triggers adaxial leaf rolling because of the reduced size of adaxial pavement cells, and an abnormal expansion of palisade mesophyll cells (Perez-Perez et al. 2010). The downstream target genes of the well-characterized TCP3 include 6 of the 29 members of the *IAA/AUX* gene family as well as *IAA3/SHY2* (Koyama et al. 2010). TCP5 and TCP17 interact with PIF4 to up-regulate the expression of *IAA19* and consequently regulate thermomorphogenesis, and TCP13 also contributes to this response (Han et al. 2019; Zhou et al. 2019). Among the 29 *IAA/AUX* genes in Arabidopsis (Bargmann et al. 2013; Weijers and Wagner 2016), *IAA5*, *IAA10*, *IAA19*, and *IAA31* are upregulated, whereas *IAA29* is downregulated by dehydration stress (Shani et al. 2017). The *IAA5, IAA6*, and *IAA19* genes are positively regulated by the DREB2A TF to promote stress-induced growth inhibition required for dehydration tolerance (Shani et al. 2017). Thus, we speculate that TCP13 might induces the growth inhibition through the dehydration-inducible auxin repressor, *IAA5* and *19* under dehydration stress.

Our results showed that TCP13 is required for the expression of *LBD1* under dehydration stress (Fig. 8) and in response to IAA treatment (Fig. 9). LBD1 is member of the ASYMMETRIC LEAVES2 (AS2)/LOB family in Arabidopsis (Iwakawa et al. 2002). AS2 forms a repressor complex with ASYMMETRIC LEAVES1 (AS1) that acts directly on class I *KNOX* genes (Guo et al. 2008), which promote stem cell activity and must be repressed to form determinate lateral organs (Jackson et al. 1994; Long et al. 1996). AS2 also forms a protein complex with CIN-like TCPs, including TCP2, 3, 4, 10, and 24, to repress the *KNOX* genes (Li et al. 2012). *TCP3* transcriptionally activates *AS1* expression to repress *CUC* genes that causes the repression of *KNOX* genes (Aida et al. 1999; Hibara et al. 2003; Koyama et al. 2010). Additionally, AS2 forms a trimetric complex with AS1 and another LBD protein, JAGGED LATERAL ORGANS (JLO), to coordinate auxin distribution and meristem function through the regulation of *PIN* and *KNOX* expression in shoots and roots (Rast and Simon 2012). Overexpression of the poplar (*Populus tremula* × *Populus alba*) *LBD1* ortholog (*PtaLBD1*) enhances secondary phloem production in populus. Overexpression of *PtaLBD1* downregulates the expression of *KNOX* genes, *ARK1* and *ARK2*, involved in vascular cambium maintenance (Yordanov et al. 2010). The negative regulation of *KNOX* genes by CIN-like TCPs in a CUC-dependent and -independent manner is the core process underlying the promotion of differentiation of leaves (Koyama et al. 2010). Based on these reports, our results support the idea that TCP13 is required for the dehydration-inducible *LBD1* expression, which potentially affects the leaf development in response to dehydration stress.

Although an increase in ABA level and signaling has long been recognized as an inhibitor of primary root growth (Antoni et al. 2013; Fujii et al. 2007; Gonzalez-Guzman et al. 2012; Park et al. 2009; Yoshida et al. 2010), knowledge about the underlying mechanisms is incomplete. Interestingly, *tcp13* mutant plants were insensitive to the ABA treatment and showed greater root growth than WT plants (Fig. 4). Additionally, *35Spro::TCP13OX* transgenic seedlings showed reduced root growth compared with control plants (Fig. 3), and this reduction was dramatically enhanced upon ABA treatment (Fig. 4). Moreover, *TCP13pro::TCP13SRDX* transgenic seedlings showed greater root growth than control plants under normal growth conditions (Fig. 3). Our transcriptome analysis showed that genes containing both ABRE and TCP binding motifs in their promoter regions were significantly downregulated in *TCP13pro::TCP13SRDX* plants (Fig. 7). The expression of ABA-regulated genes such as *AHG3*, *RD20*, *NCED3*, and *GolS2* was reduced in *tcp13* mutant plants under dehydration stress (Fig. 8) and in response to ABA treatment (Fig. 9). These results suggest that TCP13 might acts downstream of ABA signaling and participates in the inhibition of root growth under stress conditions. Several factors are involved in root growth inhibition via the ABA signaling pathway, with auxins being of prime importance. Auxin accumulation, distribution, transport, and signal transduction significantly affect primary root development (Overvoorde et al. 2010; Petricka et al. 2012; Sun et al. 2018). In Arabidopsis, ABA reduces the auxin level in roots, resulting in root growth arrest (Sun et al. 2018). ABA, dehydration, and PEG treatments prominently attenuate the response of roots to auxin in transgenic plants expressing auxin sensor constructs such as *DR5::GUS*, *IAA2pro::GUS*, and *IAA19pro::Venus* (Shani et al. 2017; Wang et al. 2011; Yang et al. 2014). High concentrations of ABA decrease the expression of genes encoding auxin transporters, such as AUX1 and PINs, in roots (Promchuea et al. 2017; Yang et al. 2014). ABI5 also suppresses *PIN1* expression, and the *abi5* mutant exhibits enhanced auxin transport in roots (Yuan et al. 2014). These results suggest that ABA inhibits root development by impacting auxin transport and signaling. Our transcriptome analysis showed that expression levels of *IAA5*, *IAA19*, and *PIN4* were reduced in *TCP13pro::TCP13SRDX* (Fig. 7) and *tcp13* mutant plants under normal growth or dehydration stress conditions (Fig. 8). Based on these reports, our results support the idea that TCP13 probably contributes the root growth inhibition under dehydration stress through negative regulation of auxin signaling.

In conclusion, the present study proposes that TCP13 might act as a positive regulator of dehydration stress tolerance through regulation of ABA and auxin signaling genes. Our results suggest that the dehydration-inducible TCP13 performs unique functions in regulating plant growth to cope with dehydration stress, and facilitates the ABA signaling. Hypothetical model of molecular framework of TCP13 under dehydration stress is presented in Figure S8. Thus, the results of this study enhance our understanding of the molecular mechanisms regulating plant growth and stress tolerance in response to dehydration stress. However, additional experiments are necessary to elucidate the detailed molecular mechanisms, especially direct regulation of ABA signaling and auxin signaling genes by TCP13. Future investigations should determine how TCP13 affect the ABA and auxin signaling genes in leaves and roots and what factors regulate the plant growth and stress tolerance through TCP13 regulation.

## Supplementary Information

Below is the link to the electronic supplementary material.

Supplementary file1 (PDF 225 kb)

Supplementary file2 (PDF 3217 kb)

Supplementary file3 (XLSX 13 kb)

Supplementary file4 (XLSX 61 kb)

Supplementary file5 (XLSX 13 kb)

Supplementary file6 (XLSX 451 kb)

## Data Availability

Microarray design and data were deposited at ArrayExpress (https://www.ebi.ac.uk/arrayexpress/) (Accession No. E-MTAB-9336).
