# Correction to: CIN‑like TCP13 is essential for plant growth regulation under dehydration stress

**DOI:** 10.1007/s11103-022-01251-2

**Published:** 2022-02-24

**Authors:** Kaoru Urano, Kyonoshin Maruyama, Tomotsugu Koyama, Nathalie Gonzalez, Dirk Inzé, Kazuko Yamaguchi-Shinozaki, Kazuo Shinozaki

**Affiliations:** 1 Gene Discovery Research Group, RIKEN Center for Sustainable Resource Science (CSRS), 3-1-1 Koyadai, Tsukuba, Ibaraki 305-0074 Japan; 2 Present Address: Institute of Agrobiological Sciences, NARO 3-1-3 Kannondai, Tsukuba, Ibaraki 305-8604 Japan; 3 Plant Biotechnology Division, Japan International Research Center for Agricultural Sciences (JIRCAS), 1-1 Ohwashi, Tsukuba, Ibaraki 305-8686 Japan; 4 Bioorganic Research Institute, Suntory Foundation for Life Sciences, Seikacho, Kyoto 619-0284 Japan; 5 INRAE, Université de Bordeaux, UMR1332 Biologie du Fruit Et Pathologie, 33882 Villenave d’Ornon Cedex, France; 6 Department of Plant Biotechnology and Bioinformatics, Ghent University, 9052 Ghent, Belgium; 7 VIB Center for Plant Systems Biology, 9052 Ghent, Belgium; 8 Laboratory of Plant Molecular Physiology, Graduate School of Agricultural and Life Sciences, The University of Tokyo, Bunkyo-ku, Tokyo, 113-8657 Japan

## Correction to: Plant Molecular Biology (2022) 108:257–275 10.1007/s11103-021-01238-5

The article CIN-like TCP13 is essential for plant growth regulation under dehydration stress, written by Urano, K., Maruyama, K., Koyama, T. et al., was originally published Online First without Open Access. After publication in volume 108, issue 3, page 257–275 the author decided to opt for Open Choice and to make the article an Open Access publication. Therefore, the copyright of the article has been changed to © The Author(s) 2022 and the article is forthwith distributed under the terms of the Creative Commons Attribution 4.0 International License, which permits use, sharing, adaptation, distribution and reproduction in any medium or format, as long as you give appropriate credit to the original author(s) and the source, provide a link to the Creative Commons licence, and indicate if changes were made.

The images or other third party material in this article are included in the article’s Creative Commons licence, unless indicated otherwise in a credit line to the material. If material is not included in the article’s Creative Commons licence and your intended use is not permitted by statutory regulation or exceeds the permitted use, you will need to obtain permission directly from the copyright holder.

To view a copy of this licence, visit http://creativecommons.org/licenses/by/4.0

