# Supplementary material for: CIN-like TCP13 is essential for plant growth regulation under dehydration stress
Source: Plant Mol Biol. 2022 Jan 20;108(3):257–75. doi: 10.1007/s11103-021-01238-5 (PMC8873074; doi:10.1007/s11103-021-01238-5)
Supplement: Supplementary file 1 — Supplementary file1 (PDF 225 kb) [file 11103_2021_1238_MOESM1_ESM.pdf]

Supporting Experimental Procedures

Plasmid construction for transient expression assays

Effector plasmids containing *AREB1* and *AREBΔQT* used in transient transactivation assays were generated previously (Fujita *et al.*, 2005). The bZIP DNA-binding domain constructs were generated by cloning PCR-amplified partial or whole *AREB1* cDNA into the *Not*I sites of the expression vector *pBI35SΩ*. The resulting construct (*pBI35SΩ-AREB1*) was partially digested with *Eco*T14I and then self-ligated to remove a 0.64 kb fragment. The resultant plasmid, *pBI35SΩ-AREB1ΔQT*, carried an internal deletion (193–831 bp) spanning the Q to T region. The *pGKX-TCP13* effector construct has been described in Experimental Procedures (under the "Plasmid construction and plant transformation" section). The *AFP2* was amplified by PCR using sequence-specific primers, and cloned into the *Eco*RV site of the *pGKX* vector, as described for *pGKX-TCP13*. The *RD29Bpro-GUS* (Uno *et al.*, 2000) and *pBI35S-LUC* (Fujita *et al.*, 2005) reporter plasmids were kindly provided by Dr. Yasunari Fujita (JIRCAS).

Transient expression assays using Arabidopsis protoplasts

Transient expression assays using protoplasts derived from Arabidopsis leaf mesophyll cells were performed as described previously (Kidokoro *et al.* 2009, Yoo *et al.* 2007). Leaves of 6-week-old plants were cut into 0.5–1 mm sections using a clean razor blade. The leaf sections were digested in 25 mL of an enzyme solution (Celllase Onozuka R10 and Macerozyme R10 (Yakult)) by vacuum infiltration for 15 min and then incubated in the dark at 22–25°C for 3.5 h. The isolated protoplasts were washed and suspended at a concentration of approximately 1.2×10^5 cells mL^{-1}. Then, 3–5 µg each of the effector (*pBI35SΩ-AREB1*, *pBI35SΩ-AREB1ΔQT*, and *pGKX-TCP13*) and reporter (*RD29Bpro-GUS*) constructs were co-transfected with 3 µg of the internal control plasmid (*pBI35S-LUC*) into the protoplasts using polyethylene glycol (PEG) solution containing 40% (w/v) PEG 4000 (Fluka; http://www.sigma-aldrich.co.jp/fluka/first.htm), 0.2 M mannitol, and 100 mM $Ca(NO_3)_2$. The transfected protoplasts were incubated under 25 µmol m^{-2} s^{-1} light intensity for more than 16 h. LUC activity was assayed using the Bright-Glo Luciferase Assay System (Promega; http://www.promega.co.jp/). Both the luminescence and fluorescence intensities were measured with a multilabel counter (Wallac AVROsx 1420; Perkin-Elmer; http://www.perkinelmer.co).

Gene-specific primers for quantitative real-time PCR (qRT-PCR) analysis

The following primers were used for qRT-PCR: *AHG3*-F 5′-CCGCTTGAAAACTGCCGTAA-3′ and *AHG3*-R 5′-CCGGTAACGTCGAGAGCACTA-3′; *FLS1*-F 5′-TCACAACATTCCGAGGTCCAA-3′ and *FLS1*-R 5′-GTCGGGATCGCTTAGATCGA-3′; *Gols2*-F 5′-CACCGTACCGGTCCACTCA-3′ and *Gols2*-R 5′-TTCCAGCAAGGAAAGTCACGTA-3′; *IAA5*-F 5′-GTCCATCTCCGGGAAGAAGAG-3′ and *IAA5*-R 5′-CGCCGGTTCACATTTCAAAT-3′; *IAA19*-F 5′-TGGCCACCGGTTTGTTCTT-3′ and *IAA19*-R 5′-TGGCCACCGGTTTGTTCTT-3′; *LBD1*-F 5′-CACCGCGGGTAGTGCTTAG-3′ and *LBD1*-R 5′-GCACAACGTCGCCTCAAGAT-3′; *LOX2*-F 5′-AGTGCCATGCATCCCATTTATAG-3′ and *LOX2*-R 5′-

TGCACGAGCGTTGATTTCC-3′; *NCED3*-F 5′-CCAGATTGCTTCTGCTTCCAT-3′ and *NCED3*-R 5′-GACCCTATCACGACGACTTCATC-3′; *PIN4*-F 5′-CCGTGGCGCTAAGCTTCTTA-3′ and *PIN4*-R 5′-CGAAACAATAGACGCACCAGTCT-3′; *TCP5*-F 5′-CGTCTGCCGGAGATGGAT-3′ and *TCP5*-R 5′-TGTCGGGAATAGAGAGCTCATTG-3′; *TCP13*-F 5′-TGGTTGCTTGATGCAGCTAAA-3′ and *TCP13*-R 5′-GAAATTTTCCGGCGAGATAGG-3′; *TCP17*-F 5′-CCGGCGGCGGAAAC-3′ and *TCP17*-R 5′-GAAGTTGACCACCACCGAGAA-3′; *RD20*-F 5′-TTAGCTCCGGTCACCAGTCA-3′ and *RD20*-R 5′-ATGTATGGTTTTGGTAATGTTTCC-3′ and *At2g32170*-F 5′-ACTGCGCTCCACATGAGAGA-3′ and *At2g32170*-R 5′-GGCCGGCTGACAAGAAGAG-3′.

References

Fujita, Y., Fujita, M., Satoh, R., Maruyama, K., Parvez, M.M., Seki, M., Hiratsu, K., Ohme-Takagi, M., Shinozaki, K. and Yamaguchi-Shinozaki, K. (2005) AREB1 is a transcription activator of novel ABRE-dependent ABA signaling that enhances drought stress tolerance in Arabidopsis. *Plant Cell*, **17**, 3470-3488.

Kidokoro, S., Maruyama, K., Nakashima, K., Imura, Y., Narusaka, Y., Shinwari, Z.K., Osakabe, Y., Fujita, Y., Mizoi, J., Shinozaki, K. and Yamaguchi-Shinozaki, K. (2009) The phytochrome-interacting factor PIF7 negatively regulates DREB1 expression under circadian control in Arabidopsis. *Plant Physiol*, **151**, 2046-2057.

Yoo, S.D., Cho, Y.H. and Sheen, J. (2007) Arabidopsis mesophyll protoplasts: a versatile cell system for transient gene expression analysis. *Nat Protoc*, **2**, 1565-1572.
