# Supplementary material for: CIN-like TCP13 is essential for plant growth regulation under dehydration stress
Source: Plant Mol Biol. 2022 Jan 20;108(3):257–75. doi: 10.1007/s11103-021-01238-5 (PMC8873074; doi:10.1007/s11103-021-01238-5)
Supplement: Supplementary file 2 — Supplementary file2 (PDF 3217 kb) [file 11103_2021_1238_MOESM2_ESM.pdf]

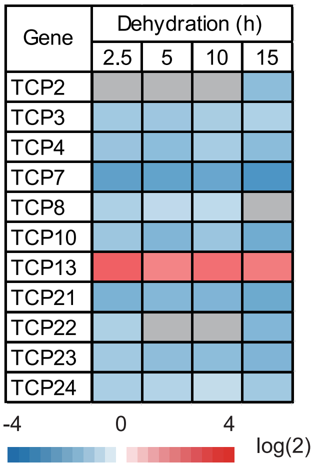

Figure S1. Microarray analysis of temporal changes in the expression patterns of Arabidopsis *TCP* genes.

The heat map shows ratios of gene expression in wild-type (WT) plants under dehydration conditions relative to that under control conditions. Gene expression data were obtained from (Urano *et al*. 2009). Unidentified data are indicated in gray.

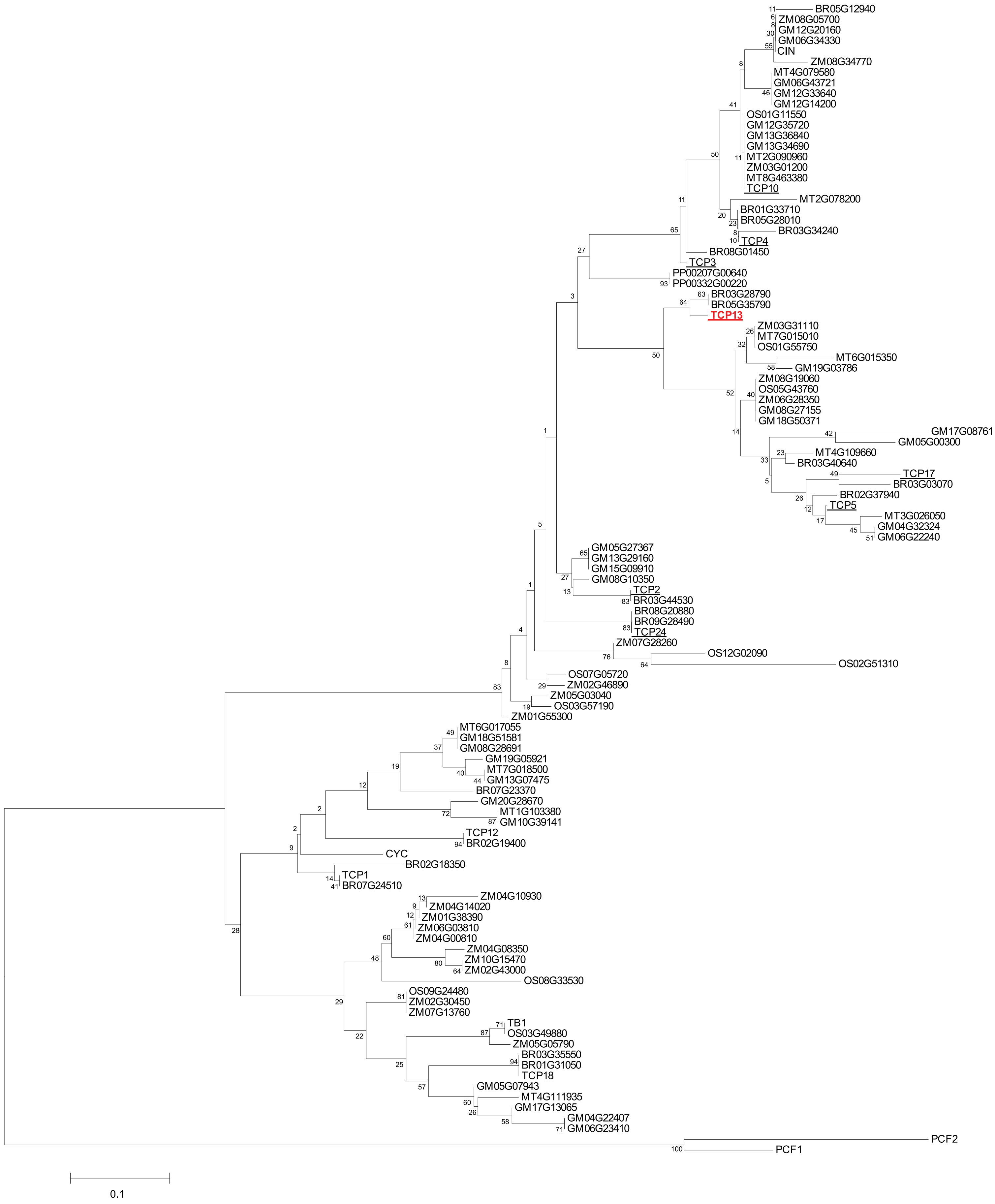

Figure S2. Phylogenetic analysis of dicot, monocot and moss class II TCPs.

The phylogenetic tree of dicot and monocot class II TCPs and representative members of CINCINNATA (CIN) and CYCLOIDEA (CYC) families of Antirrhinum majus was constructed using the neighbor-joining method with the MEGA X software. Rice (Oryza sativa) class I TCPs, PROLIFERATING CELL FACTOR1 (PCF1) and PCF2 were used as an outgroup. Arabidopsis CIN-like TCPs were indicated by an underline and TCP13 indicates by a red color.

(a) *TCP13* expression data in mesophyll cell from EFP browser

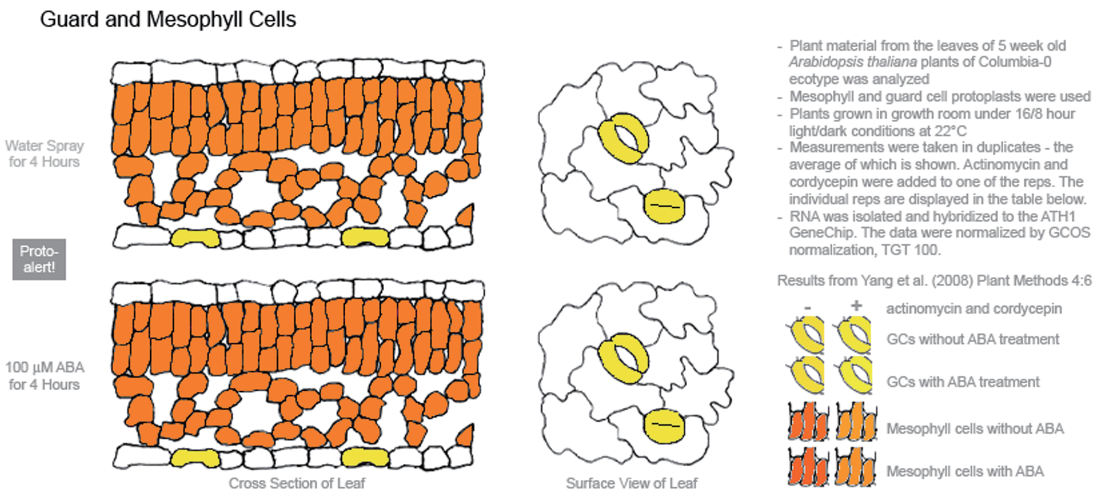

(b) *TCP13* expression data in roots from EFP browser

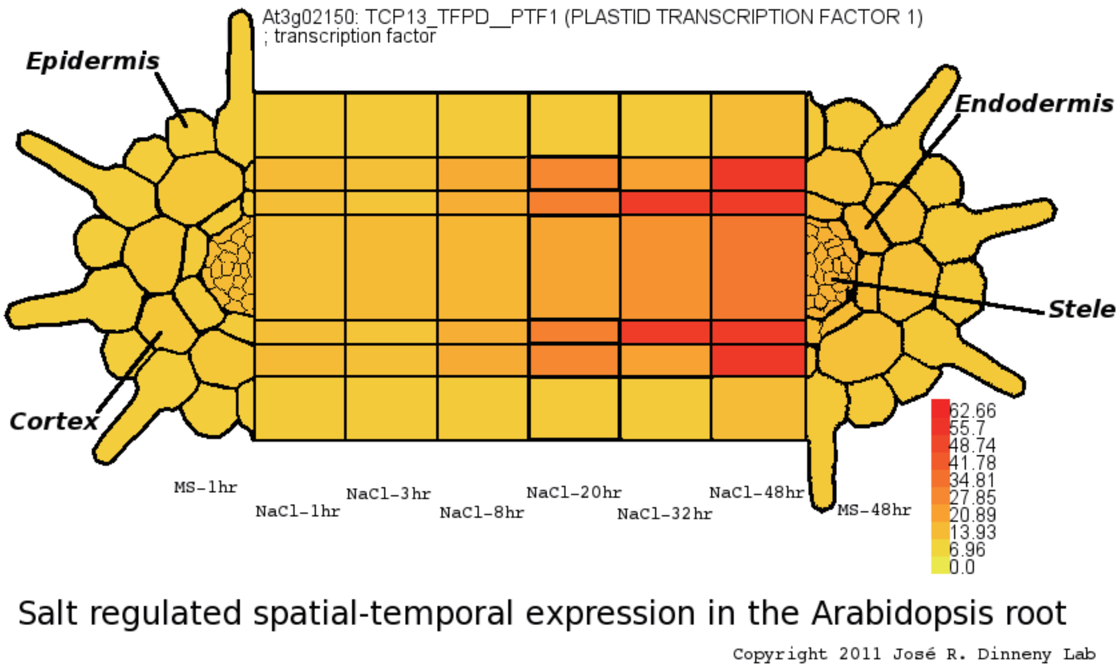

Figure S3. Expression analysis of *TCP13* in leaves and roots of Arabidopsis plants using publicly available data.
(a and b) Expression of *TCP13* in leaves, particularly mesophyll cells (a), and in roots, particularly in endodermis and cortex cells (b) under salt stress. Tissue-specific *TCP13* expression data obtained from the Arabidopsis EFP browser (http://bar.utoronto.ca/efp/cgi-bin/efpWeb.cgi).

(a)

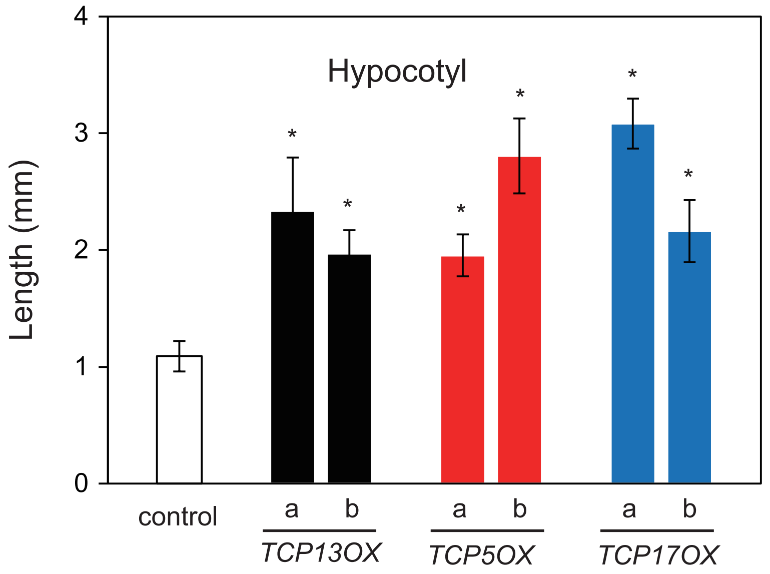

(b)

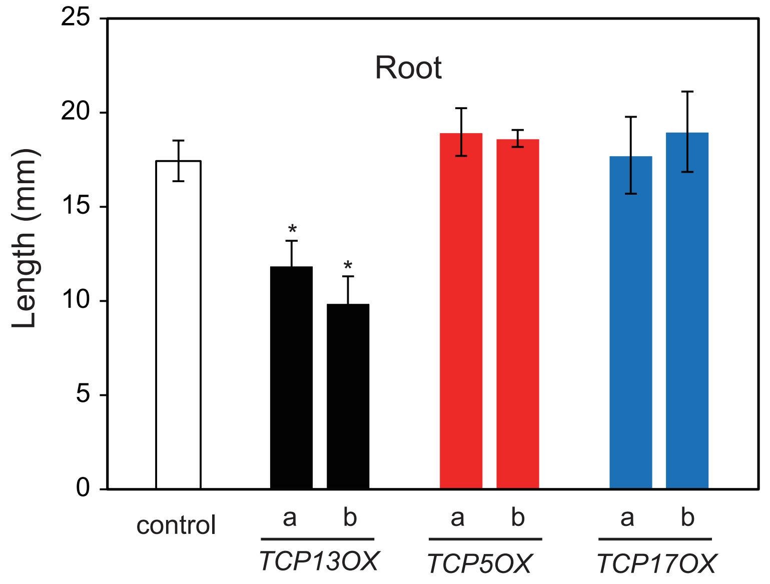

Figure S4. Hypocotyl and root lengths of transgenic Arabidopsis plants overexpressing *TCP5*, *TCP13*, or *TCP17* under the control of the constitutive 35S promoter.

(a and b) Hypocotyl (a) and root (b) lengths of 7-day-old vector control, *35Spro::TCP5OX*, *35Spro::TCP13OX*, and *35Spro::TCP17OX* seedlings. Data represent mean ± SD (n = 6).

An asterisk shows that the indicated mean is significantly different from the mean value of the control plant (**P* < 0.05, ; one-way ANOVA with Welch’ s *t* test).

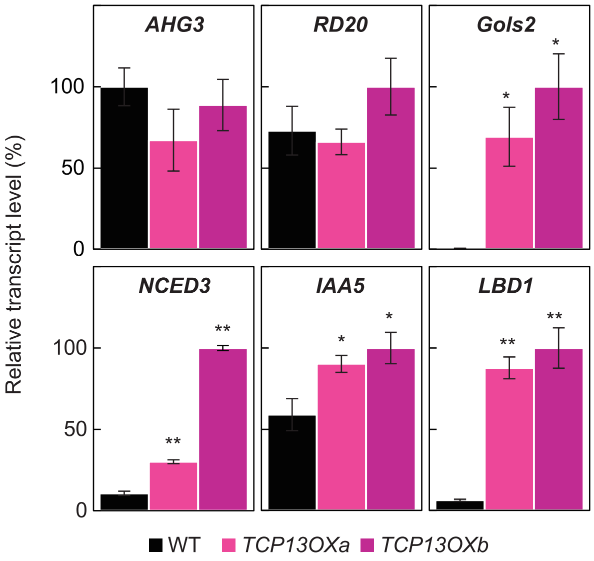

Figure S5. Expression analysis of downstream target genes of TCP13 in *35Spro::TCP13OX* plants by qRT-PCR. In each case, the maximum gene transcript level was set to 100. Data represent mean ± SD of technical replicates (n = 3). An asterisk shows that the indicated mean is significantly different from the mean value of the control plants (*$P < 0.05$, **$P < 0.01$, one-way ANOVA with Welch's *t* test).

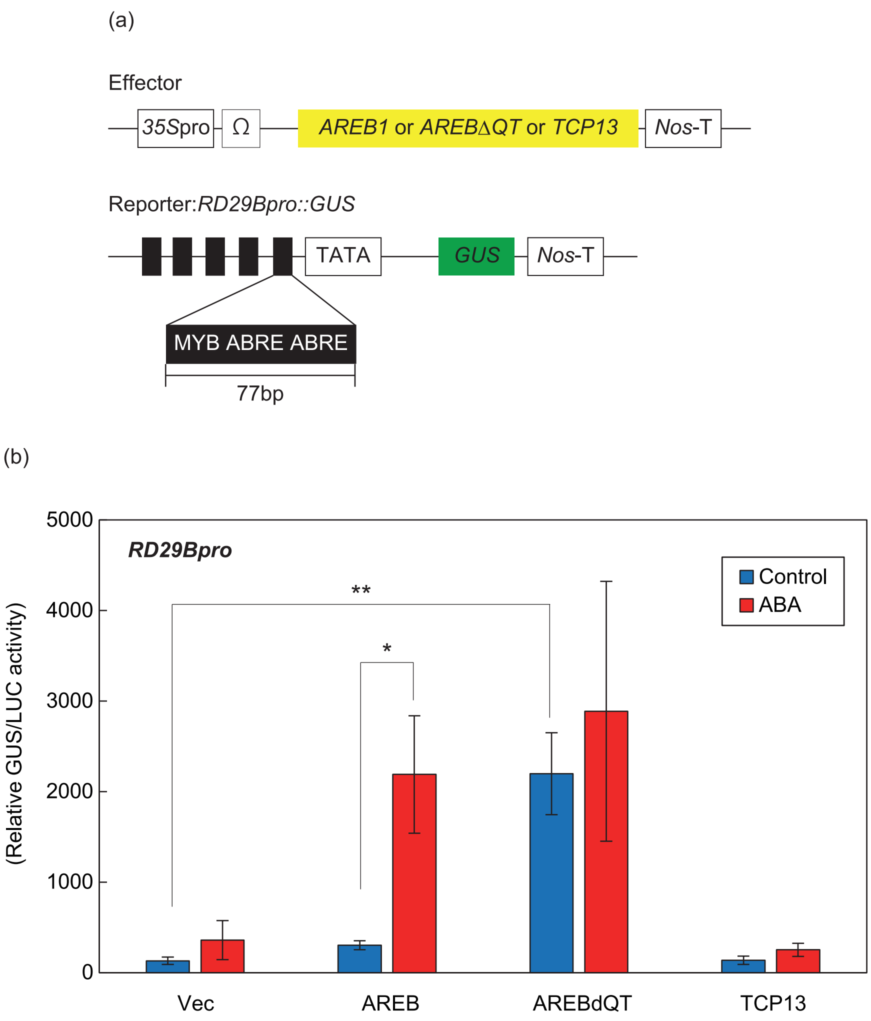

Figure S6. Analysis of the activation of the *RD29B* by TCP13.

(a) Schematic of the effector and reporter constructs used in transactivation assays. The effector constructs contained the Cauliflower mosaic virus (CaMV) 35S promoter and tobacco mosaic virus sequence fused to *AREB1*, active form of *AREB1* (*AREB1ΔQT*), or *TCP13* cDNA fragments. The *RD29B* promoter *GUS* reporter construct (*RD29Bpro::GUS*) contained five tandem repeats of a 77 - bp fragment of the *RD29B* promoter fused to *GUS* construct and *nopaline synthase* terminator (*Nos*-T). The transactivation activity of *AREB1*, *AREB1ΔQT*, and *RD29B* promoter constructs was reported previously (Fujita *et al*. 2005).

(b) Transactivation analysis of *AREB1*, *AREB1ΔQT*, or *TCP13* with the *RD29B*. Protoplasts were co - transfected along with the *RD29Bpro::GUS* and an effector construct, and treated with or without 10 μM ABA. To normalize for the transfection efficiency, the luciferase (LUC) reporter construct (*pBI35SΩ- LUC*) was co - transfected as a control in each experiment, and the GUS/LUC activity was calculated. Data represent mean ± SD of three technical replicates.

An asterisk shows that the indicated mean is significantly different from the mean value of the control construct under the corresponding condition (**P* < 0.05, ***P* < 0.01, one-way ANOVA with Welch’ s *t* test).

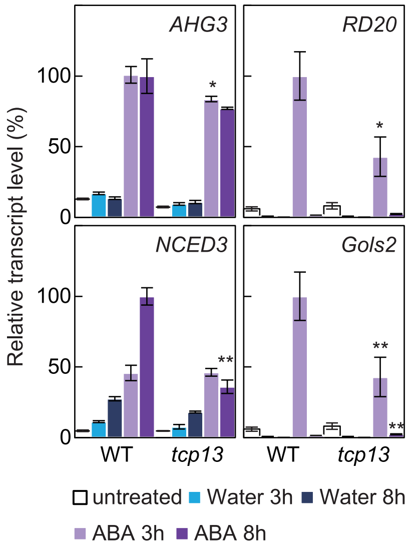

Figure S7. Expression analysis of downstream target genes of TCP13 in ABA-treated *tcp13* mutant plants by qRT-PCR. ABA- and dehydration-inducible genes analyzed by qRT-PCR. In each case, the maximum gene transcript level was set to 100. An asterisk shows that the indicated mean is significantly different from the mean value of the wild-type plant under the corresponding condition (*$P < 0.05$, **$P < 0.01$, one-way ANOVA with Welch' s *t* test).

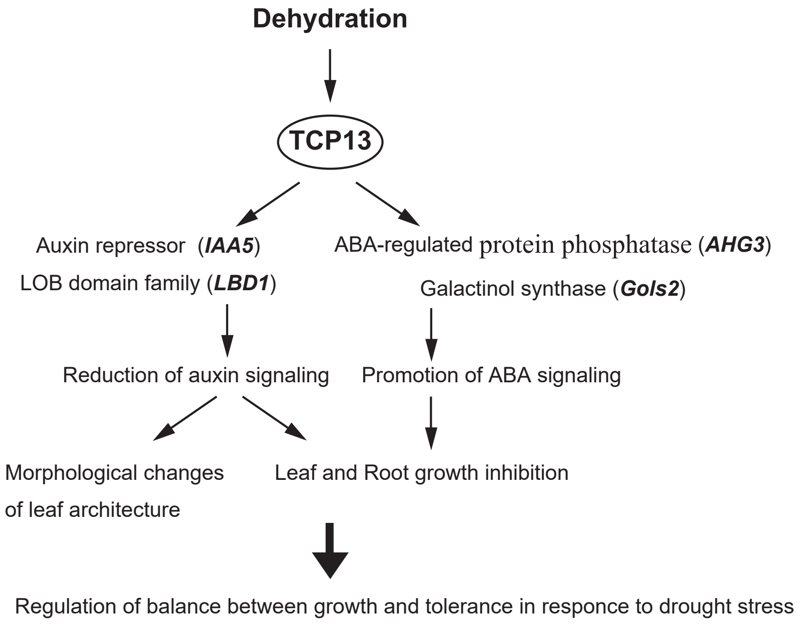

Figure S8. Hypothetical model of the molecular framework of TCP13 under dehydration stress.
Dehydration-inducible TCP13 regulates auxin-regulated genes, including auxin repressor (*IAA5*) and LOB domain family gene (*LBD1*), leading to morphological changes of leaf architecture and leaf and root growth regulation to enhance dehydration stress tolerance. TCP13 also helps to facilitate ABA-regulated genes, including ABA-hypersensitive Germination3 (*AHG3*) and galactinol synthase (Gols2). TCP13-dependent transcriptional regulatory network controls not only genes involved in stress tolerance, but also genes involved in plant growth in response to drought stress.
